# Global changes in gene expression during compatible and incompatible interactions of cowpea (*Vigna unguiculata* L.) with the root parasitic angiosperm *Striga gesnerioides*

**DOI:** 10.1186/1471-2164-13-402

**Published:** 2012-08-17

**Authors:** Kan Huang, Karolina E Mellor, Shom N Paul, Mark J Lawson, Aaron J Mackey, Michael P Timko

**Affiliations:** 1 Department of Biology, University of Virginia, Gilmer Hall 044, Charlottesville, VA, 22904, USA; 2 Center for Public Health Genomics, University of Virginia School of Medicine, Charlottesville, VA, 22908, USA

**Keywords:** Cowpea, Defense, Resistance, *Striga gesnerioides*, Transcription, *Vigna unguiculata*, Witchweed

## Abstract

**Background:**

Cowpea, *Vigna unguiculata* L. Walp., is one of the most important food and forage legumes in the semi-arid tropics. While most domesticated forms of cowpea are susceptible to the root parasitic weed *Striga gesnerioides*, several cultivars have been identified that show race-specific resistance. Cowpea cultivar B301 contains the *RSG3-301* gene for resistance to *S. gesnerioides* race SG3, but is susceptible to race SG4z. When challenged by SG3, roots of cultivar B301 develop a strong resistance response characterized by a hypersensitive reaction and cell death at the site of parasite attachment. In contrast, no visible response occurs in B301 roots parasitized by SG4z.

**Results:**

Gene expression in the roots of the cowpea cultivar B301 during compatible (susceptible) and incompatible (resistant) interactions with *S. gesnerioides* races SG4z and SG3, respectively, were investigated at the early (6 days post-inoculation (dpi)) and late (13 dpi) stages of the resistance response using a Nimblegen custom design cowpea microarray. A total of 111 genes were differentially expressed in B301 roots at 6 dpi; this number increased to 2102 genes at 13 dpi. At 13 dpi, a total of 1944 genes were differentially expressed during compatible (susceptible) interactions of B301 with SG4z. Genes and pathways involved in signal transduction, programmed cell death and apoptosis, and defense response to biotic and abiotic stress were differentially expressed in the early resistance response; at the later time point, enrichment was primarily for defense-related gene expression, and genes encoding components of lignifications and secondary wall formation. In compatible interactions (B301 – SG4z), multiple defense pathways were repressed, including those involved in lignin biosynthesis and secondary cell wall modifications, while cellular transport processes for nitrogen and sulfur were increased.

**Conclusion:**

Distinct changes in global gene expression profiles occur in host roots following successful and unsuccessful attempted parasitism by *Striga*. Induction of specific defense related genes and pathways defines components of a unique resistance mechanism. Some genes and pathways up-regulated in the host resistance response to SG3 are repressed in the susceptible interactions, suggesting that the parasite is targeting specific components of the host’s defense. These results add to our understanding of plant-parasite interactions and the evolution of resistance to parasitic weeds.

## Background

Cowpea (*Vigna unguiculata* L. Walp.) is the most important grain legume grown in sub-Saharan Africa [1,2]. Approximately 12.5 million tons of cowpea grains are produced worldwide each year, with the majority of the production (over 64%) taking place on low-input, subsistence farms in west and central Africa [3]. In these regions, cowpea is often referred to as “poor man’s meat” because of its high protein content (20-25%) and good nutritional value [4]. The fruits are consumed at all stages of growth (e.g., green pods, fresh or dry seeds) and the young leaves are often used for soups and stews [5]. In addition to its value as human food, cowpea hay is an important source of animal fodder [6]. Two characteristics add to the plant’s agronomic importance: it is generally drought tolerant and it fixes nitrogen symbiotically, thereby enhancing soil fertility, especially when used in rotation with cereals [7].

Like most crops, cowpea growth and grain yields are greatly reduced by a variety of biotic pests (e.g., bacterial, fungal and viral diseases; insects; nematodes; herbivores) and biotic stresses (severe drought, salinity, and heat) [2]. Among the major biotic constraints is parasitism by *Striga gesnerioides* L Walp. (Orobanchaceae), commonly referred to as witch weed. Witch weed is a noxious and persistent pest in farm fields: yield losses due to *S. gesnerioides* parasitism are extensive in the Sudano-Sahelian belt of west and central Africa [8]. Control of the parasite is difficult because it produces thousands of seeds per generation that remain in the seed bank for years and also because most of the damage to its host plant occurs prior to its emergence from the ground [9]. The damaging effects of *Striga* in this region are further compounded by poor soils and drought.

While most cowpea plants are susceptible to *Striga* parasitism, some local landraces and wild accessions have been identified that are resistant and in most reports, resistance is a dominant characteristic, inherited in a monogenic manner [2,10]. Complicating the identification of *Striga*-resistant germplasm is the variable nature of the parasite with at least seven distinct races of *S. gesnerioides* [designated SG1 (Burkina Faso), SG2 (Mali), SG3 (Nigeria and Niger), SG4 (Benin), SG4z (Zakpota region of Benin), SG5 (Cameroon), and SG6 (Senegal)] now identified throughout West Africa [11-13]. Analysis of several advanced populations -- segregating for resistance to one or more of the different races of *S. gesnerioides* has resulted in the genetic mapping of several race-specific resistance (R) genes within the cowpea genome and the development of molecular markers linked to these genes [14]. Using a positional cloning approach, Li and Timko [14] isolated and characterized a gene (designated *RSG3-301*) capable of conferring resistance to *S. gesnerioides* race 3 (SG3). *RSG3-301* encodes an R protein homolog containing a coiled-coil (CC) protein-protein interaction domain at the N-terminus, a nucleotide binding site (NBS), and a leucine-rich repeat domain at the C-terminus. Silencing of *RSG3-301* expression in the resistant cultivar B301 leads to susceptibility to race SG3, but does not affect resistance to other races of the parasite, underscoring the specificity of the resistance response [14].

**Figure 1:**
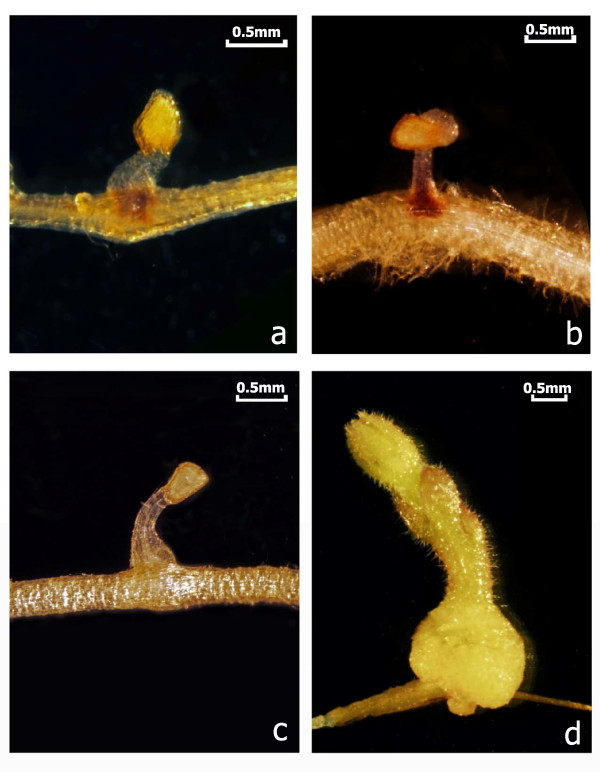
**Representative photographs showing the differential response of roots of cowpea cultivar B301 to infection by two different races of *S. gesnerioides. *** (**A**) Roots infected with *S. gesnerioides* race SG3 at 6 days post-inoculation (dpi) showing a hypersensitive response; (**B**) Roots infected with *S. gesnerioides* race SG3 at 13 dpi showing a hypersensitive response and parasite death; (**C**) Roots infected with *S. gesnerioides* race SG4z at 6 dpi showing early penetration of host; (**D**) Roots infected with *S. gesnerioides* race SG4z at 13 dpi showing parasite growth.

Resistant cowpea genotypes exhibit two different response mechanisms to *Striga* attack. When challenged by a known race, cultivars carrying the appropriate race-specific resistance gene exhibit a rapid and robust hypersensitive response (HR) typified by browning and necrosis at the site of parasite attachment, and subsequent rapid death of the parasite within 3-4 days [13,15,16] (Figure 1). In host plants lacking the appropriate resistance gene, the parasite rapidly penetrates the host root cortex, forms connections to the host vascular system, swells to form a tubercle, and expands its cotyledons leading to subsequent above ground growth and flowering.

To date, only limited information is available on transcription changes in hosts in response to *Striga* parasitism [17-19]. To address this gap in our knowledge and to better understand the mechanics of host resistance to parasitic weeds, we have investigated the nature of global gene expression changes that occur during compatible and incompatible *S. gesnerioides* -cowpea interactions, using a custom designed cowpea Nimblegen microarray. These studies have identified candidate genes and potential pathways involved in resistant and susceptible responses to attempted *Striga* parasitism which can be tested further to gain additional information on this unique plant-plant interaction.

## Results

### Design of cowpea microarray platform

Whole genome microarrays provide a means to access gene expression involved in a particular cellular or developmental event on a global scale. Since a microarray platform was not commercially available for cowpea at the time of this study, we designed a 385,000 feature long-nucleotide custom microarray to interrogate 43,253 cowpea unigenes of known and unknown function, previously identified in a gene-space sequencing study [20,21]. On the fabricated microarray, each predicted gene coding region is represented by six to eight 60-mer oligonucleotide probes.

To validate our microarray design, we examined the distribution of mean probe set intensity and probe set variance over all assayed samples ( Additional file 1) to identify problematic probe sets that failed to hybridize (extremely low mean and variance) or hybridized nonspecifically (extremely high mean and low variance). The results of this analysis showed that of 49,287 probe sets on the microarray, 20,224 probe sets (41%) exhibited intensity profiles consistent with target-specific RNA hybridization. High intra-probe set covariance between individual probe intensities indicate that 75% of probe sets were composed of functional probes whose variation in intensities robustly represented abundance of the intended target RNA species ( Additional file 2).

### Investigating global changes in gene expression during compatible and incompatible cowpea-*Striga* interactions

To examine the response of cowpea roots to attempted parasitism by *S. gesnerioides*, we used cowpea cultivar B301 as our host plant. Cowpea cultivar B301 is a land-race from Botswana that was originally identified as resistant to *Striga* in field studies and subsequently shown to be resistant to all races of *S. gesnerioides* known at the time [22]. However, in the early 1990s, a new hyper virulent race of the parasite (SG4z), identified in the Zakpota region of Benin, was discovered to overcome the resistance present in B301 [11]. B301 still maintains its resistance to all other races outside of this area. The differential response of B301 parasite races provides a good model for examining global changes in gene expression in the same genetic background during compatible/susceptible (B301-SG4z) and incompatible (B301-SG3) interactions (Figure 1). For our analysis, samples from three biological replicates were collected at two time points (6 dpi and 13 dpi). These time points were chosen on the basis of previous histological studies indicating that the earliest evidence of a hypersensitive response can be found at 6 dpi, with complete host cortical root browning and necrosis of the parasite evident by 13 dpi [13]. Conversely, by 13 dpi of compatible interaction, host-parasite vascular connections have been established, and tubercle swelling and parasite cotyledon expansion have initiated.

Differential expression analysis was conducted as described in the Materials and Methods section, and genes were identified as differentially expressed if the adjusted P value was less than or equal to 0.05 (i.e. a 5% false discovery rate [FDR] threshold). Based on these criteria, at 6 dpi, the expression of 111 genes was significantly altered in B301 roots infected with *S. gesnerioides* race SG3 (resistant interaction) compared to uninfected control roots (Table 1). At 13 dpi, the number of significantly altered genes increases dramatically to 2102, among which 52 are genes whose expression was also altered at the earlier 6 dpi time point ( Additional file 3). A greater proportion (54%) of the differentially regulated genes were upregulated at both the early and late stages in the incompatible interactions. The large increase in the number of differentially expressed genes for the late time point of the resistant response is not unexpected since while activation of a defense cascade leading to the resistant phenotype and its associated hypersensitive response (i.e., browning, apoptosis) is rapid, propagation of the response and subsequent biosynthetic and physiological changes are broader and more persistent.

**Table 1:** **Number of genes differentially regulated in the various cowpea- *Striga *interactions **

**Interaction**	Up-regulated genes*	Down-regulated genes*	Total*
SG3 6 dpi (incompatible)	81	30	111
SG3 13 dpi (incompatible)	1111	991	2102
SG4z 13 dpi (compatible)	855	1089	1944

Given in the table the number of significantly (*p < 0.05) up- and down-regulated genes and the total number of differentially regulated genes in interactions of cowpea B301 roots with *Striga* races SG3 and SG4z.

In compatible interactions of B301 with SG4z, 1944 genes had their expression altered compared to control roots at 13 dpi (Table 1). The expression of the majority (1089/1944) of these genes was down-regulated compared to uninfected control roots. Of the differentially regulated genes found, 923 genes were common in both resistant and susceptible interactions at 13 dpi (Additional file 3). There were 1179 and 1021 genes that were unique in the resistant and susceptible interactions, respectively.

The fold-change ratios, adjusted P values, and most significant GenBank annotations for selected genes from the various treatments are presented in Tables 2, 3, and 4. The genes were chosen based on the assumption that genes with the highest fold-change ratio are most likely to have a direct role in the plant-parasite interaction.

**Table 2:** **Representative genes significantly up- and down-regulated in roots of cowpea cultivar B301 6 days post-inoculation with *S. gesnerioides *race SG3 **

**Category**	**Sequence ID**	**Annotation**	**Fold change**	***P-*value**
*Transcription factors*	33693912	POZ/BTB containing-protein AtPOB1	5.57	0.003
	33693491	asymmetric leaves 1, phantastica (AS1)	5.25	0.027
	33670861	zinc finger (RING-H2 type) protein-related	4.8	0.003
	33684347	basic helix-loop-helix (bHLH) DNA-binding	3.27	0.019
*Secondary metabolism*	33658828	chalcone synthase	2.03	0.050
*Signal transduction*	33685813	serine carboxypeptidase-like 23 (scpl23)	12.75	0.000
	33673870	5'-AMP-activated protein kinase beta-2 subunit	3.57	0.040
	33649427	cytochrome P450 (CYP722A1)	2.57	0.006
	33684072	CDKE1 (Cyclin-dependent kinase E1)	2.41	0.016
*Cellular transport*	33666738	aluminum activated malate transporter	17.62	0.006
	33676680	folate transporter 1 (FOLT1)	4.15	0.036
*Defense related*	33646015	chitinase 1 precursor, narbonin	44.57	0.000
	33690846	cationic peroxidase 1 precursor	5.18	0.005
	33662461*	class III peroxidase	3.83	0.004
	33663803	class III peroxidase	3.71	0.039
	33663380	beta galactosidase7 (BGAL7)	3.13	0.048
	33651959	1,3-beta-D-glucanase	2.84	0.007
	33680262	disease resistance protein (TIR-NBS-LRR class) family	1.91	0.015
*Plant growth regulators*	33646791	jasmonate-zim domain protein 1 (JAZ1)	2.23	0.039
*Cell component*	33692312	copper ion-binding laccase	4.41	0.039
	33677650	laccase	3.8	0.042
	33686210	pectin methylesterase	3.24	0.011
*Unknown protein*	33675717	unnamed protein product	12.86	0.023
	33657146	unnamed protein product	4.78	0.040
*Unknown protein*	33684891	expressed protein contains Pfam profile PF03140	-8.83	0.000
	33657530	expressed unknown protein	-4.52	0.037
*Metabolism*	33686080	C2 domain-containing protein similar to phloem protein	-6.54	0.000
	33681048	ferric reductase	-4.39	0.009
*Transcription*	33667792	aluminum-activated malate transporter	-4.9	0.036
*Cell cycle, DNA processing*	33677672	RAD51A recombination protein	-4.52	0.042

*genes verified by qRT PCR.

**Table 3:** **Representative genes significantly up- and down-regulated in roots of cowpea cultivar B301 13 days post-inoculation with *S. gesnerioides *race SG3**

**Categories**	**Sequence ID**	**Annotation**	**Fold change**	***P *value**
*Transcription factors*	33693912	POZ/BTB containing-protein AtPOB1	6.04	0.000
	33646298	chlorophyll a/b-binding protein CP24 precursor	5.2	0.012
	33693491*	asymmetric leaves 1 (AS1), phantastica	4.57	0.000
	33683206	growth regulating factor 4 (GRF4)	4.53	0.006
	33654115	ethylene responsive element 3 (ERF3)	4.08	0.010
	33681428*	RING-H2 finger protein	2.91	0.006
*Energy*	33666738	ALMT1 (Aluminum activate malate transporter)	19.62	0.001
	33676680	folate transporter 1 (FOLT1)	4.23	0.005
	33679899	ABC transporter-like protein	2.85	0.047
*Cellular transduction*	33675942*	Ser/Thr protein kinase	10.98	0.015
	33668608	Leucine-rich repeat protein kinase family	6.62	0.002
	33684252	FU (FUSED); protein serine/threonine kinase	3.97	0.023
	33647807	serine/arginine rich protein	3.18	0.027
	33671868	receptor-like protein kinase 2	3.04	0.001
*Defense*	33646015	chitinase 1 precursor, narbonin	6.06	0.000
	33663380	beta-galactosidase	4.21	0.003
	33667640	disease resistance protein RPM1	2.15	0.044
*Development*	33668608	leaf senescence-associated receptor kinase	6.62	0.001
	33653025	auxin-regulated protein	3.67	0.008
	33645492	ripening related protein (MLP43)	3.05	0.014
*Cellular component*	33686210	pectin methylesterase	3.29	0.002
	33650922	4-coumarate – CoA-lipase	3.23	0.030
*Unknown protein*	33675717	unnamed protein product	4.27	0.050
	33658803	hypothetical protein	3.71	0.015
*Unknown protein*	33669403	predicted protein	-12.21	0.001
	33684891	hypothetical protein	-10.8	0.000
	33691084	predicted protein	-8.36	0.000
	33660796	predicted protein	-7.24	0.001
*Cellular transport*	33672142	nodulin MtN21 family protein	-11.55	0.000
	33657530	early nodulin 55-2 precursor	-7.55	0.006
	33689578	oligopeptide transporter OPT family	-7.24	0.004

*genes verified by qRT PCR.

**Table 4:** **Representative genes significantly up- and down-regulated in roots of cowpea cultivar B301 13 days post-inoculation with *S. gesnerioides *race SG4z **

**Category**	**Sequence ID**	**Annotation**	**Fold change**	***P *value**
*Defense related*	33692796	class 5 chitinase	-18.83	0.035
	33650557	lipoxygenase	-9.92	0.000
	33650653	disease resistance response protein	-9.84	0.005
	33681057	peroxidase	-9.63	0.001
	33691436	pectate lyase	-8.86	0.002
	33674444	acidic chitinase	-6.62	0.000
	33664131	dehydration-responsive family protein	-6.5	0.000
	33692483	peroxidase	-6.08	0.000
	33680968	peroxidase precursor	-5.67	0.004
	33648572	peroxidase precursor	-4.33	0.002
	33681614	cytochrome P450 monooxygenase	-4.19	0.000
	33681310	ATP dependent copper transporter	-2.89	0.03
	33688073	phenylalanine ammonia lyase (PAL1)	-2.06	0.04
*Plant Growth Regulator*	33669665	auxin efflux carrier protein	-4.48	0.000
	33654433	auxin-regulated protein	-3.99	0.003
	33656035	auxin-regulated protein (Aux28)	-3.85	0.002
	33672768	auxin efflux carrier protein 10	-3.85	0.024
	33673426	IAA14	-3.41	0.001
	33677822	auxin-responsive-like protein	-3.14	0.001
	33685840	gibberellin 2-oxidase	-2.52	0.021
	33692861	auxin influx carrier protein	-2.45	0.005
	33656492	aux/IAA protein	-2.4	0.029
*Cellular components*	33654029	expansin	-17.12	0.000
	33672947	expansin	-7.53	0.001
	33679526	glucosyltransferase	-3.88	0.000
	33688119	beta-expansin	-3.85	0.020
	33651745	cellulose synthase-like protein CslG	-3.01	0.022
	33676145	peptidase C14, caspase catalytic subunit p20	-2.5	0.029
*Cellular transport*	33678156	multifunctional aquaporin	4.29	0.000
	33671842	putative nitrate transporter NTL1	2.6	0.008
	33690036	amino acid permease family protein	2.37	0.014
	33673354	high affinity sulphate transporter	2.32	0.029
	33664197	sugar transporter	1.95	0.031
	33674339	putative ammonium transporter AMT2	1.61	0.028
	33653963	putative amino acid or GABA permease	1.44	0.034
*Cellular signal transduction*	33662517	stress kinase	2.37	0.003
	33659136	stress-inducible H1 histone-like protein	2.04	0.003

In the incompatible interaction between B301 and SG3 (Table 2), among the most highly up-regulated genes at 6 dpi are those encoding various transcription factors and signal transduction components, defense-related proteins (e.g., chitinase and peroxidase homologs, narbonin), and proteins involved in cell wall biogenesis. Among the most highly down-regulated genes at 6 dpi are those encoding cell cycle regulators and cellular transporters. At the later stages (13 dpi) of incompatible interactions (Table 3), up-regulated gene expression is dominated by genes encoding various transcription factors, signal transduction components, proteins involved in cellular energy metabolism and proteins associated with developmental regulation.

In contrast, in B301 roots being successfully parasitized by SG4z (Table 4), among the most highly down-regulated genes are those encoding defense-related proteins (e.g., peroxidase, chitinases), plant growth regulators (including multiple auxin transporters and auxin responsive regulators), and proteins involved in cell wall biogenesis (e.g., expansins, cellulose synthase, phenylalanine ammonium lyase and glucosyltransferase). Of the various genes up-regulated in this compatible interaction, of particular note are those encoding various nitrogen and sulfur transporters and permeases.

In order to verify that the changes in gene expression detected in our microarray analysis reflect actual changes in transcript levels, quantitative reverse-transcriptase PCR (qRT-PCR) was carried out using total RNAs samples isolated from plant tissue samples from identical treatments harvested independently. In most cases for the various genes tested by qRT-PCR, the levels of induction or repression (fold-change in expression) observed were comparable (Additional file 4).

### Co-regulated genes and pathways in incompatible cowpea B301-*S. gesnerioides* race SG3 interactions

**Figure 2:**
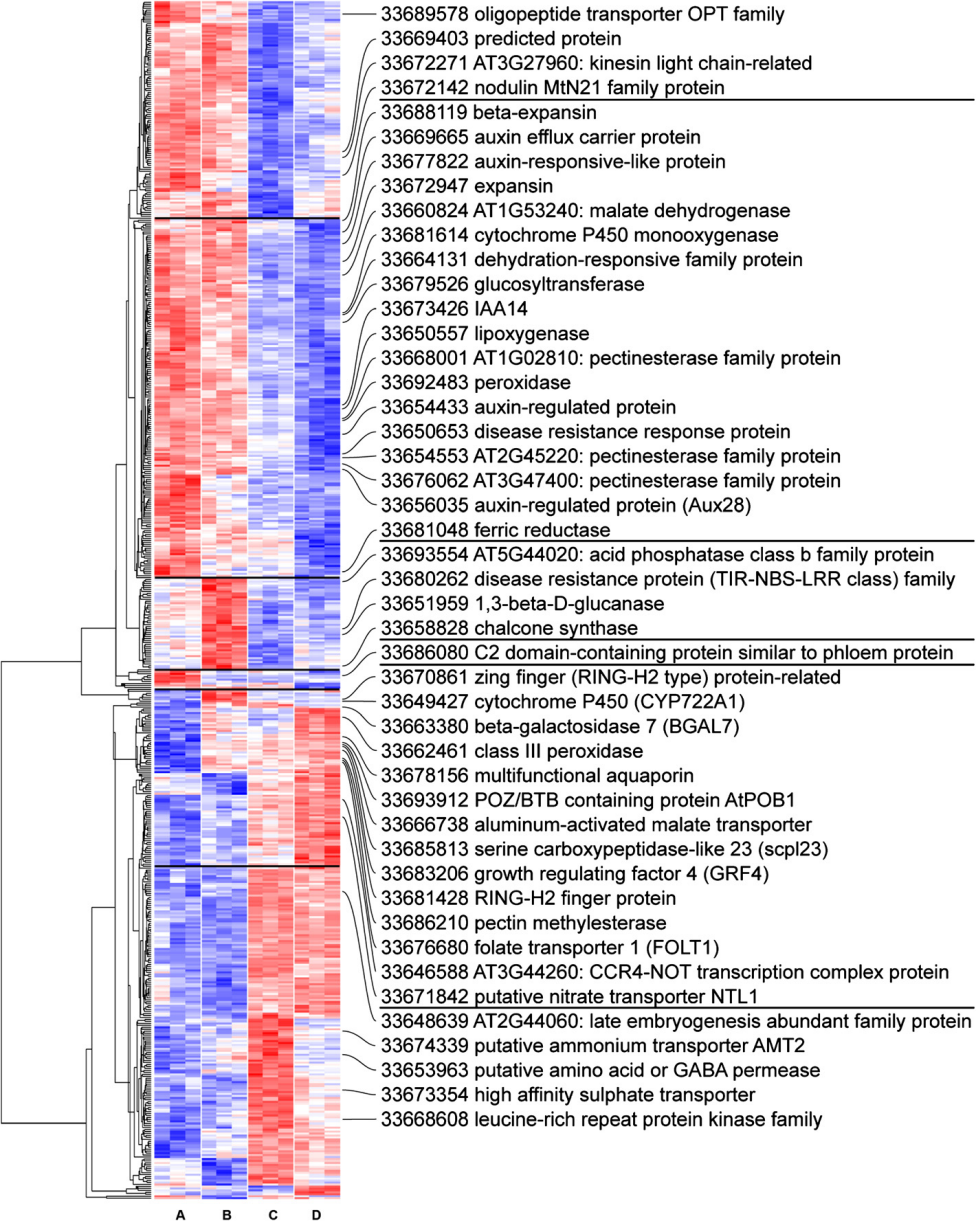
**Heatmap of normalized intensity values obtained from cowpea microarray at 0.1% FDR threshold. ** Material collected represent resistant and susceptible cowpea-* Striga * interaction at early (6 dpi) and late (13 dpi) stage of *Striga* attachment. Genes were clustered based on their intensity values profile across treatments and expressed as 6 groups linked by a dendrogram. Representative genes from these various clusters on the heatmap are labeled on the side. Each treatment (A = control, B = SG3 6 dpi, C = SG3 13 dpi, D = SG4z) consisted of three independent biological replicas. Genes with relatively high intensity values versus all treatments are marked red. Genes with relatively low intensity values compared across treatments are marked blue. Genes with average intensities compared to other treatments are marked white.

To identify genes and pathways that are co-regulated within the various treatments, differentially expressed genes were clustered based on their intensity values profile across treatments and expressed as a colored grid (heatmap), with genes ordered by a dendrogram representing covariance across treatments (Figure 2, Additional file 5). Several clusters of co-regulated genes can be identified within replicates of early (6 dpi) and late (13 dpi) incompatible interactions, and within compatible B301-SG4z interactions. Representative genes from these various clusters on the heatmap that show a full expression profile at the 0.1% FDR threshold are depicted in Additional file 6.

**Figure 3:**
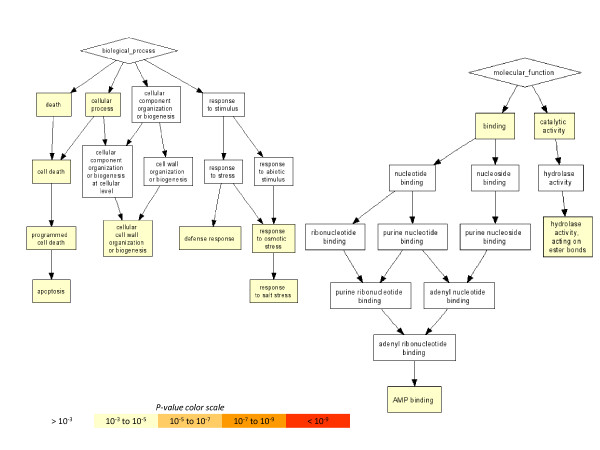
**GOterm gene enrichment. ** Enrichment was generated using Gorilla with a P-value less than 10^-3^ using differentially expressed GSRs at a 5% FDR threshold in cowpea infected with *S.gesnerioides* race SG3 at early stage of infection (6 dpi).

We used GOrilla (Gene Ontology enRIchment anaLysis and visuaLizAtion tool) to identify pathways enriched (P-values less than 10^-3^) with differentially expressed genes in the early (6 dpi) incompatible interaction between B301 and SG3 (Figure 3, Additional file 7). Among the biological processes showing the most significant changes in gene expression were pathways associated with cell death, programmed cell death, and apoptosis, as well as pathways associated with response to stress and biotic stress. We also see significant enrichment of differentially expressed genes associated with cell wall biogenesis and organization, and genes categorized under molecular processes, such as hydrolase activity and catalytic activity. Enrichment in these categories is not unexpected. One of the key features of incompatible interactions between cowpea and *S. gesnerioides* is the elicitation of a hypersensitive response when host roots expressing the appropriate race-specific resistance gene are challenged by a parasite with a recognized avirulence factor, such as in the case of B301 parasitized by *S. gesnerioides* race SG3 [14].

**Figure 4:**
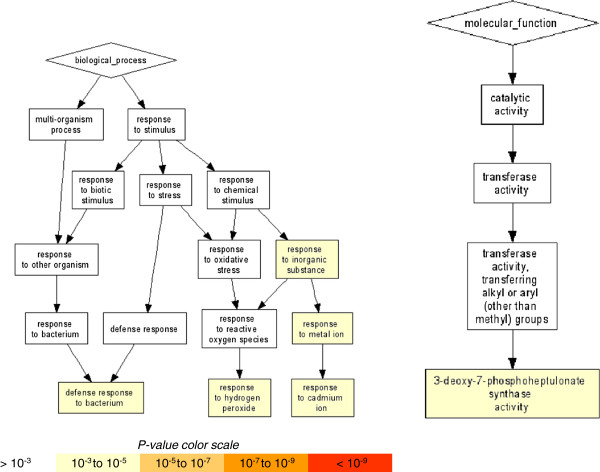
**GOterm gene enrichment. ** Enrichment was generated using Gorilla with a P-value less than 10^-3^ using differentially expressed GSRs at a 5% FDR threshold in cowpea infected with *S. gesnerioides* race SG3 at late stage of infection (13 dpi).

During the late stages of the B301-SG3 incompatible interaction (13 dpi), defense pathways associated with response to a variety of biotic stresses are among the biological processes showing the most significant changes in gene expression (Figure 4, Additional file 8). Significant alteration to gene expression was also seen in pathways involved in response to oxidative stress, signaling pathways for secondary metabolism, and pathways involved in multiple plants’ biosynthetic and chemical detoxification processes.

### Co-regulated genes and pathways in compatible cowpea B301-*S. gesnerioides* race SG4z interactions

**Figure 5:**
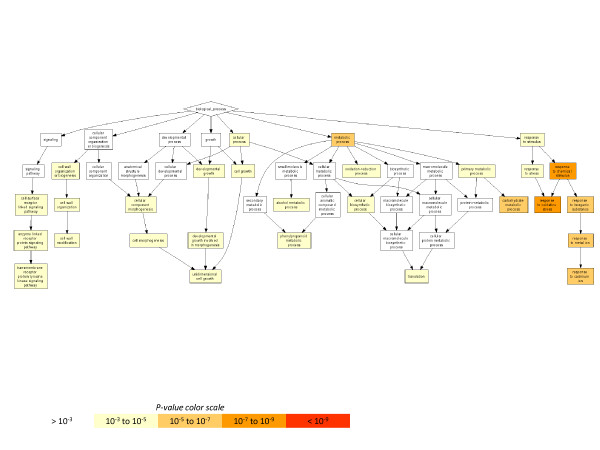
**GOterm gene enrichment. ** Enrichment was generated using Gorilla with a P-value less than 10^-3^ using differentially expressed GSRs at a 5% FDR threshold in cowpea infected with *S.gesnerioides* race SG4z at late stage of infection (13 dpi).

In striking contrast to the response of B301 roots to SG3, B301 roots parasitized by SG4z show no phenotypic manifestation during parasite ingress through the cortex. Nonetheless, dramatic alterations in gene expression are elicited with the greatest enrichment of differential expression found in pathways associated with cellular differentiation and growth, cell signaling and metabolism, and defense signaling (Figure 5, Additional file 9). These observed changes in cellular function are consistent with the dramatic down-regulation of gene expression involved in auxin transport and signaling, both critical for cellular growth and proliferation, and down-regulation of the expression of genes involved in cell wall growth (e.g., expansins) and reinforcement (e.g., enzymes of cellulose, lignin, and callus formation).

As it might be expected, B301 roots that were actively parasitized by SG4z also had gene expression differences enriched in pathways associated with defense signaling and response to biotic and abiotic stress. The active suppression of defense response cascades by pest- and pathogen-derived effectors that attack host cellular targets is well characterized; the global alterations in signaling and stress response cascades observed in our studies indicate that similar activities are likely in play in these host-parasite associations.

## Discussion

### Gene expression in incompatible *Striga*-host interactions

The mechanics of plant host response to attempted parasitism and the nature of the gene expression changes occurring during both compatible and incompatible interactions between *S. gesnerioides* and cowpea have been investigated using oligonucleotide-based microarrays. Up until now, only limited information was available on global changes in transcription taking place in hosts in response to *Striga* parasitism. Our data show that alterations, both positive and negative, in a wide range of processes occur as a consequence of *Striga* attack. In cultivars harboring resistance genes capable of interfacing with a defense signal transduction pathway, as in the case of cowpea cultivar B301 which contains multirace resistance to *S. gesnerioides*, a rapid and robust defense response is elicited in the form of hypersensitivity at the site of parasite attachment, under conditions where the invading parasite is recognized. We found that accompanying the visible hypersensitive response is the up-regulation of genes involved in signal transduction and biosynthetic processes associated with formation barriers to prevent parasite ingress. These include activation of gene expression associated with cell wall biogenesis and strengthening (e.g., lignifications), as well as processes leading to programmed cell death at the host-parasite interface.

In a study of the interaction of *S. hermonthica* with its host plant sorghum*,* Hiraoka and Sugimoto [17] observed that attempted parasitism induced JA-responsive genes and suppressed SA-responsive genes in the roots of a highly susceptible cultivar, and suggested that susceptible hosts recognize *Striga* parasitism as wounding stress rather than microbial stress. In contrast, they found that in the roots of a moderately resistant sorghum cultivar, both SA- and JA-responsive genes were induced suggesting that resistance involved pathways associated with both wounding and pathogen challenge. Pretreatment of the roots of the moderately resistant sorghum cultivar with SA led to an inhibition of *Striga* development suggesting that the SA-responsive genes are directly involved in host resistance mechanism. In a subsequent study, subtractive hybridization was used to examine the interaction of *Lotus japonicus* with *P. aegyptiaca* (a compatible interaction) and *S. hermonthica* (incompatible) [23]. These investigations found little or no overlap among the *Phelipanche* and *Striga*-induced transcripts, suggesting that *L. japonicus* roots are able to distinguish the compatible parasite from the incompatible one. Among the genes specifically induced by *P. aegyptiaca* were those encoding components of JA biosynthesis, whereas *S. hermonthica* parasitism induced genes in phytoalexin biosynthesis.

Examination of the differential expression of genes encoding candidate disease resistance and signal transduction components during the interactions of *S. gesnerioides* with various cowpea cultivars revealed that PR-5 transcript levels were dramatically elevated in the roots of cowpea genotypes resistant to *Striga* compared to uninfected roots and roots challenged with a race of *Striga* to which it was susceptible or adapted to another host species [18]. In contrast, transcript levels of *COI1* and *EDS1* increased during susceptible and non-host interactions but were unchanged during resistance response. The *COI1* gene product plays a pivotal role in activation of JA-mediated response cascades and, in some cases, serves as an inhibitor of SA signaling. *EDS1* encodes lipase-like proteins that control defense activation and programmed cell death in plants. Induction of *COI1* gene expression in the compatible *Striga*-cowpea interaction suggests that *COI1* may down-regulate or suppress the resistance response and block SA signaling pathway in cowpea plants, thus allowing *Striga* attachment and further development.

Swarbrick et al. [19] characterized gene expression changes in the roots of the *S. hermonthica* resistant and susceptible rice cultivars Nipponbare and IAC 165 using a rice microarray. These investigators found that the levels of a large number of transcripts in rice roots were either positively (up-regulated) or negatively (down-regulated) affected by parasitism. Among the genes up-regulated in the *Striga*-resistant cultivar Nipponbare were ones encoding HR protein homologs; PR-proteins associated with microbial pathogenesis including endochitinases (PR-3), glucanases (PR-2), and thaumatin-like proteins (PR-5); pleiotropic drug resistance ABC transporters; and enzymes in phenylpropanoid metabolism. In addition, transcripts encoding several WRKY transcription factors (TFs), previously implicated in other SA-dependent resistance responses [24], were observed to be more abundant in parasitized roots. Large-scale down-regulation of gene expression was observed in the susceptible cultivar IAC 165, particularly transcripts whose encoded products annotate to Gene Ontology (GO) functional categories of plant growth regulator signaling and metabolism, biogenesis of cellular components, and cell division. Interestingly, a majority of the genes down-regulated following attempted *Striga* parasitism in both IAC165 and Nipponbare roots encodes products that annotate as proteins of unknown function.

A recent study that used whole genome oligonucleotide arrays to examine the transcriptome of *A. thaliana* roots after inoculation with pre-germinated *S. hermonthica* seeds found that large numbers of genes involved in cell wall synthesis, defense signaling, regulation of transcription and protein synthesis, oxidative stress, and primary and secondary metabolism were up-regulated at the earliest stages of parasite infection [25]. This included up-regulation of many genes (*EDS1, EDS5*, *PAD3*, *NPR1*, *NIMIN1*, *PR2*) involved in the SA signaling pathway, as well as the up-regulation of a key WRKY transcription factor (AtWRKY70) that regulates the expression of genes involved in the SA signaling pathway and is thought to have a role in determining the balance between SA and JA signaling. In addition, there was evidence for the activation of genes involved in the JA and ethylene biosynthetic pathways.

In the present study of cowpea-*S. gesnerioides* interactions, among the most highly induced genes in the early resistance response (6 dpi) of B301 to SG3 are those involved in response to biotic stimuli, abiotic stimuli, wounding, oxidative stress and HR, and components of the JA and ETH signaling pathways. Among the genes most highly induced were a number of chitinases and chitinase homologs including narbonin, a protein with glycosyl hydrolase activity [26] whose biological function has yet to be elucidated [27]. Narbonin has been previously suggested as interfering with parasite growth in the root by altering cell wall extensibility [19].

Lignifications and callused deposition have been previously observed at the interface of host plant cells with invading *Orobanche* haustorium [28] and is thought to be correlated with levels of host resistance to *Orobanche*. We similarly observed an increased expression of genes involved in cell wall biogenesis suggesting that lignifications may be part the mechanism of defense employed by cowpea against *S. gesnerioides* ingress.

A number of cytochrome P450s, previously implicated in various cellular detoxification pathways in plants, were also found to be induced during the resistance response of B301. Increased cytochrome P450 gene expression has been previously reported during the resistance reaction of rice infected by *S. hermonthica*[19].

The only other global study of cowpea transcriptional profiling published thus far is the work by Das et al. [29] who investigated differences in gene expression in resistant and susceptible cowpea genotypes elicited by feeding of the root-nematode, *Meloidogyne incognita*, using a soybean Affymetrix Gene Chip expression array. At 3 dpi in both compatible and incompatible interactions, more genes were down-regulated than up-regulated. When expression between infected resistant and susceptible genotypes was compared, a greater number and proportion of genes were down-regulated in the resistant than in the susceptible genotype, whereas more genes were up-regulated in the susceptible than in the resistant genotype in response to nematode infection. Gene ontology based functional categorization revealed that the typical defense response was partially suppressed in resistant roots, allowing nematode juvenile development. Das et al. [29] suggests that suppression of genes in ROS formation and other defense related responses might be important negative resistance mechanism. Our findings contrast with this, since we see activation of a wide range of processes as a consequence of *Striga* attack. Direct comparisons of gene lists suggest that in both early stages (3 dpi) of incompatible and compatible interactions there are many induced genes common in both host-parasite interactions suggesting that these may be part of a basal defense response. Among these were genes involved in lignifications and cross-linking. This response leads to heavy lignifications of cell walls and creates a mechanical barrier for the pathogen. In the incompatible cowpea-nematode interaction, expansin, thought to be important for maintaining the specialized feeding structures in hosts, was highly down-regulated. This was not observed in the cowpea-*Striga* interaction but down-regulation of expansins were observed in the response of a resistant rice cultivar to attack by *S. hermonthica*.

### Gene expression in compatible *Striga*-host interactions

We know that root parasitic angiosperms secrete pectin methylesterases, polygalacturonase, and other cell wall softening enzymes during attempted penetration of the host root cortex, and that such secretions may in fact assist in overcoming the cell wall reinforcement that occurs in the host root in response to parasite parasitism [30-33]. It is now evident that many different phytopathogens have also evolved specific effectors and virulence factors that are capable of entering the host cell and suppressing the host resistance machinery or bypassing surveillance [34-36]. Among the most highly down-regulated genes in B301 roots during a susceptible interaction with SG4z were genes in the phenylpropanoid and lignin biosynthesis pathways, primary and secondary cell wall biogenesis, and components of the SA and JA signal transduction pathways.

*S. gesnerioides*-infected cowpea often demonstrated severe reduction in the growth rate and reduction of the host biomass [37]. This was due to two major factors: direct carbon transfer from the host to *S. gesnerioides*, and reduction of photosynthetic capacity [37]. One of the noticeable changes in gene expression during the susceptible response in B301 was the suppression of plant growth regulators, particularly auxin and gibberellins. Some genes, such as cellulose synthase, known to be involved in cell wall development [38] were also observed to be down-regulated.

One of the keys to the success of a parasitic plant-host interaction is the translocation of water and nutrients (including nitrates, amino acids, carbohydrates and minerals) from host to parasite through the haustoria [32]. The identification of differentially up-regulated transcripts for transporters responsible for nitrogen, sulfur and amino acids in B301 roots during susceptible interactions with invading SG4z suggests that, in addition to suppressing some host functions to facilitate entry, the parasite is also modifying others to provide a source of nutrition. This is clearly an area that warrants additional study.

## Conclusion

Distinct changes in global gene expression profiles occur in host roots following successful and unsuccessful attempted parasitism by *Striga*. Induction of specific defense-related genes and pathways defines components of a unique resistance mechanism. Some genes and pathways up-regulated in the host resistance response to SG3 are repressed in the susceptible interactions, suggesting that the parasite is targeting specific components of host defense. These results add to our understanding of plant-parasite interactions and the evolution of resistance to parasitic weeds.

## Methods

### Plant growth and infection with *Striga gesnerioides*

Cowpea cultivar B301 seeds were surface-sterilized with 1% hypochlorite for 5 min. They were then placed between two sheets of moist glass fiber filter paper (GF/A Whatman, Piscataway, NJ), held between two blocks of moistened rockwool (Grodan Inc., Milton, ON) for 5 days and then transferred to a growth chamber, which consists of a 24 cm x 24 cm x 3 cm Petri dish containing rockwool with a 100 μm mesh separating the cowpea roots from the rockwool [14]. The chamber was covered with aluminum foil to keep light away from the roots. Cowpea seedlings were grown for another 7 days in a controlled environment growth room under a 12 H light–dark photoperiod at 30^o^C. Seeds of *S. gesnerioides* races SG3 and SG4z were surface sterilized and pre-conditioned for 9 days as previously described [13]. The germination of *Striga* seeds was triggered using root exudates from cowpea cultivar B301. The roots of each cowpea seedling were inoculated with 15 mg of pre-germinated *S. gesnerioides* using a paintbrush. Root material was collected 6 and 13 days post-inoculation (dpi). Unattached *Striga* seeds and seedlings were removed by rinsing the roots with distilled water and the infected areas on the roots were collected by cutting at 0.5 cm at either site of the infection site. Sections (1 cm long) of control roots were collected from similar positions along the root where infections occurred in *Striga* treated samples. Three biological replicates were generated for each treatment with each replicate consisting of three independently inoculated cowpea plants. Tissue samples were frozen using liquid nitrogen and stored in a -80^o^C freezer.

### RNA isolation, quantification, and hybridization to the cowpea microarray

Total RNA was isolated from frozen, finely ground root tissues as described by [39] with minor modification. RNA samples were treated with DNase (Roche Applied Science, Indianapolis, IN) and repurified using Qiagen RNeasy kit (Qiagen, Valencia, CA) according to the manufacturers’ instructions. RNA concentration and quality was determined using an Experion (Bio-Rad, Hercules, CA).

A 385,000 feature microarray was fabricated (Nimblegen Inc., Madison, WI) using 60-nucleotide long oligonucleotide synthesized based upon the nucleotide sequence of 43,253 cowpea unigenes of known and unknown function previously identified in a gene-space sequencing study [20,21]. On the fabricated microarray, each predicted gene coding region is represented by six to eight 60-mer oligonucleotide probes. For hybridization, cDNA was prepared according to the protocol provided by Roche (Roche Applied Science, Indianapolis, IN). cDNA quality control and hybridization to the 385 K arrays were performed at Nimblegen.

### Analysis of microarray data

Statistical analysis of Nimblegen microarray data was performed using software from the Bioconductor project [40] and implemented in the R environment for statistical computing and graphics (R Development Core Team, 2009), version 2.10.1.

Prior to reading the data from the raw expression values from the XYS files, an annotation package was build for the Nimblegen custom design file “2007-05-01_UVir_CowPea_expr.ndf” using the pdInfoBuilder package protocol [41] version 1.10.1. The array data was read into R using the oligo package protocol [42] version 1.10.4. RMA background subtraction and normalization were applied to the microarray set. The experiment was performed using three biological replicates.

Differential expression analysis was conducted using the Limma package, version 3.2.3. Limma analysis applies linear models to each gene and an empirical Bayesian method to moderate standard errors for estimation of fold changes. A multiple testing correction was performed using the using Benjamin-Hochberg error correction model. Genes were identified as differentially expressed if the adjusted P-value, false discovery rate (FDR) was less than 0.1.

The data discussed in this publication have been deposited in NCBI's Gene Expression Omnibus [43] and are accessible through GEO Series accession number (GSE39348, http://www.ncbi.nlm.nih.gov/geo/query/acc.cgi?acc=GSE39348).

Assembled GSR sequences were mapped against their Arabidopsis protein sequences from TAIR10 [44] using FASTX [45] version 36.06 with a P-value threshold of 10E-3. GSR sequences ordered by FDR values were analyzed for gene enrichment using GOrilla [46] with a P-value threshold of 10E-3. Assembled GSR sequences were annotated against their best hit against plant refseq [47] using FASTX with a P-value threshold of 10E-3.

Annotated and assembled GSRs were used to generate a heatmap of normalized intensity values at 0.1% FDR threshold using enhanced heatmap function in R. GSRs with significant P-values of 10E-3 have been grouped based on their intensity profile and were used to generate GO Trees using Amigo [48]. Enriched GO terms were included on the side and were matched to corresponding genes on the heatmap.

### Quantitative reverse transcriptase PCR verification of transcript levels

Transcript levels in various tissue samples were validated by qRT-PCR using new total RNA samples prepared from B301 root tissues before and following attempted *Striga* parasitism as described above. cDNAs were synthesized using the Invitrogen Thermo script Kit (Invitrogen, Caarlsbad, CA). qRT-PCR reactions were carried out using the iQ SYBR Green Super mix Kit (Bio-Rad, Hercules, CA) and gene-specific primers (Additional file 10) against selected differentially expressed target genes. Reactions were carried out on an cycler Optical Module PCR according to the manufacturer’s instructions and gene expression fold changes were calculated according to Schmittgen and Livak [49]. Expression was normalized using an actin reference gene (XM_003521168; sequence id: 33645964, GI: 356504867) giving a 132 bp amplicon. Previous studies [50] have shown that expression of this actin reference gene is constant throughout development and during interactions with *S. gesnerioides*.

## Competing interests

Authors declare that they have no competing interests.

## Authors’ contributions

MPT conceived of the project and was responsible for directing all of the research activities. KEM and KH carried out the experimental aspects of the work and assisted SNP, MJL, and AJM in computational analysis. SNP, MJL, and AJM carried out the statistical analysis of the microarray data, and worked with KEM and KH in refining gene annotations and expression analysis. All authors have assisted in the writing of the manuscript and have read and approved the final submitted version of the manuscript.

## Supplementary Material

Additional file 1 Distribution of mean probeset intensity and probeset variance. Click here for file

Additional file 2 Intraprobeset correlation. Click here for file

Additional file 3 Venn diagrams for genes up-regulated and down-regulated during resistant and susceptible interaction of cowpea with A) Striga race SG3 and B) Striga race SG4z. Click here for file

Additional file 4 Quantitative reverse-transcriptase PCR verification of differential gene expression. Click here for file

Additional file 5 Heat map 5% FDR. Click here for file

Additional file 6 Representative genes from the heatmap showing a full expression profile at 0.1% FDR threshold. Click here for file

Additional file 7 GO enrichment SG3 6 dpi. Click here for file

Additional file 8 GO enrichment SG3 13 dpi. Click here for file

Additional file 9 GO enrichment SG4z 13 dpi. Click here for file

Additional file 10 Primer sequences for qRT-PCR. Click here for file
