# Supplementary material for: Global changes in gene expression during compatible and incompatible interactions of cowpea (Vigna unguiculata L.) with the root parasitic angiosperm Striga gesnerioides
Source: BMC Genomics. 2012 Aug 17;13:402. doi: 10.1186/1471-2164-13-402 (PMC3505475; doi:10.1186/1471-2164-13-402)
Supplement: Additional file 1 — Distribution of mean probeset intensity and probeset variance. [file 1471-2164-13-402-S1.pdf]

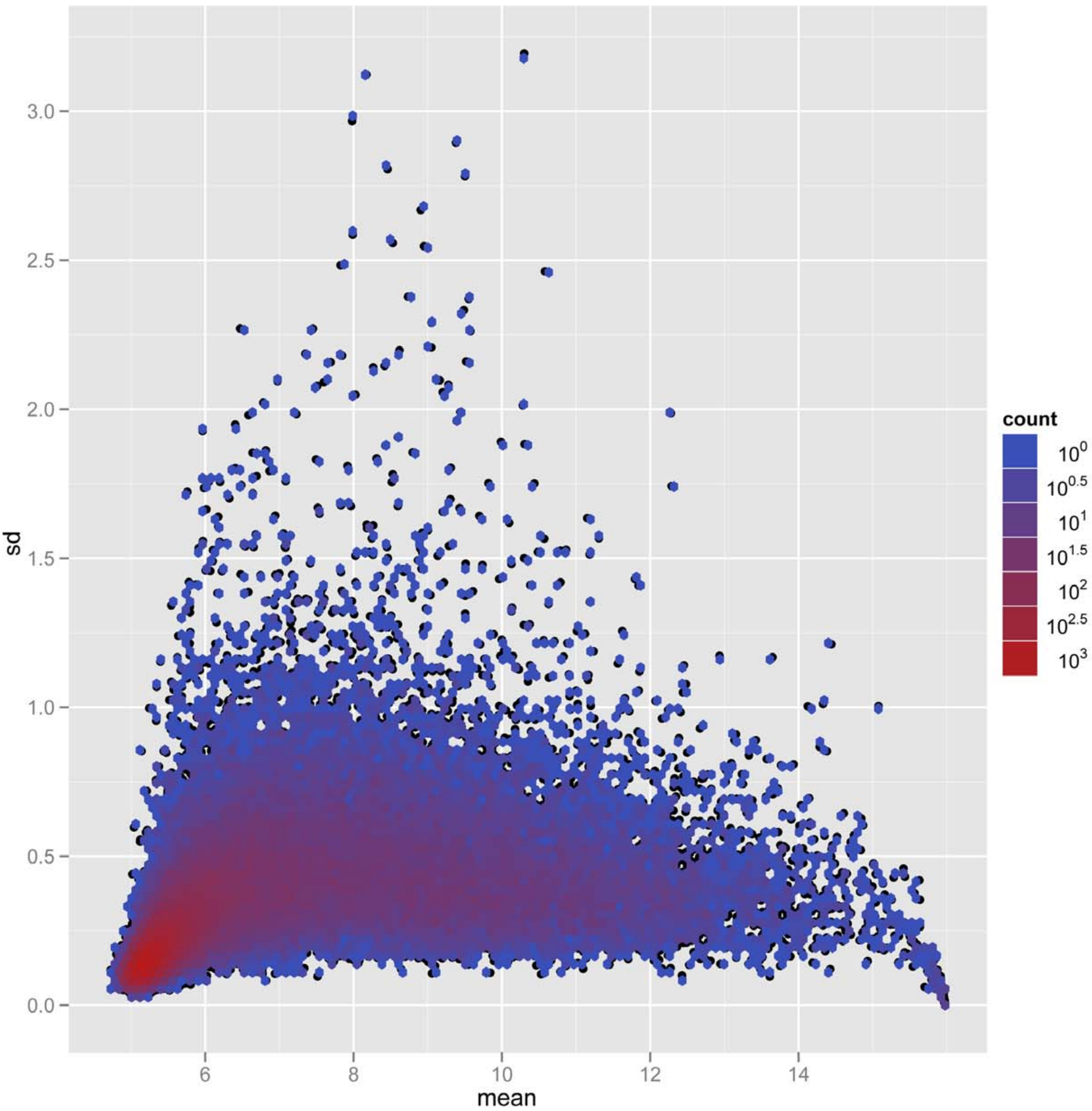

Additional file 1. Distribution of mean probeset intensity and probeset variance

Identification of problematic probesets that failed to hybridize (extremely low mean and variance) or hybridized nonspecifically (extremely high mean and low variance).
