# Supplementary material for: Global changes in gene expression during compatible and incompatible interactions of cowpea (Vigna unguiculata L.) with the root parasitic angiosperm Striga gesnerioides
Source: BMC Genomics. 2012 Aug 17;13:402. doi: 10.1186/1471-2164-13-402 (PMC3505475; doi:10.1186/1471-2164-13-402)
Supplement: Additional file 2 — Intraprobeset correlation. [file 1471-2164-13-402-S2.pdf]

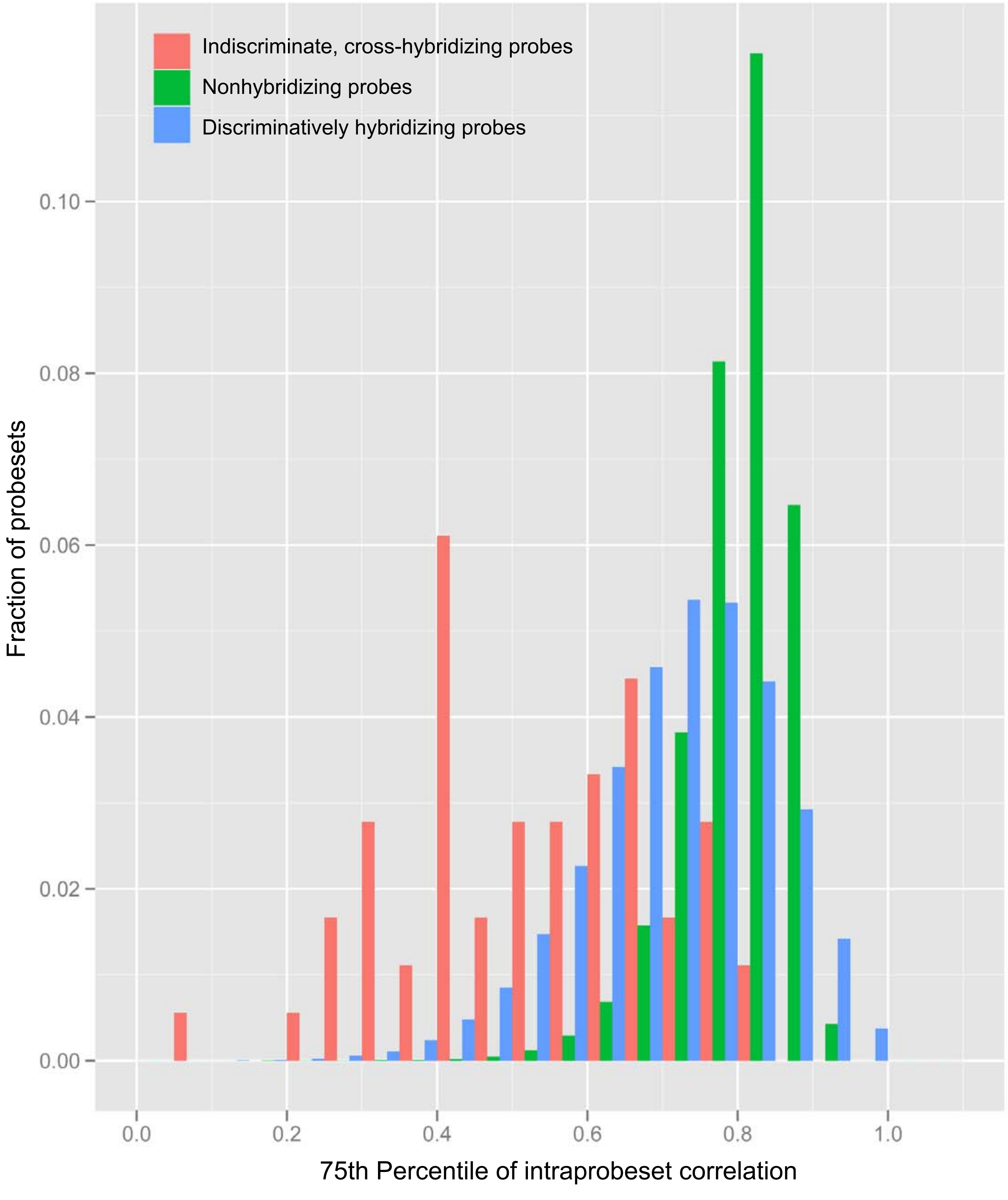

Additional file 2 Intraprobeset correlation

Degree (75th percentile) of correlation between % probe intensities within probesets that are either nonhybridizing probes (very low mean intensity, very low variation), indiscriminate, cross-hybridizing probes (very high intensity, very low variation), or otherwise discriminatively hybridizing probes showing a correlation of >0.6 for more than 75% of the various pairwise correlations.
