# Supplementary material for: Global changes in gene expression during compatible and incompatible interactions of cowpea (Vigna unguiculata L.) with the root parasitic angiosperm Striga gesnerioides
Source: BMC Genomics. 2012 Aug 17;13:402. doi: 10.1186/1471-2164-13-402 (PMC3505475; doi:10.1186/1471-2164-13-402)
Supplement: Additional file 3 — Venn diagrams for genes up-regulated and down-regulated during resistant and susceptible interaction of cowpea with A) Striga race SG3 and B) Striga race SG4z. [file 1471-2164-13-402-S3.pdf]

Additional file 3. Venn diagrams for genes upregulated and down regulated during resistant and susceptible interaction of cowpea with A) *Striga* race 3 and B) *Striga* race 4.pdf

A

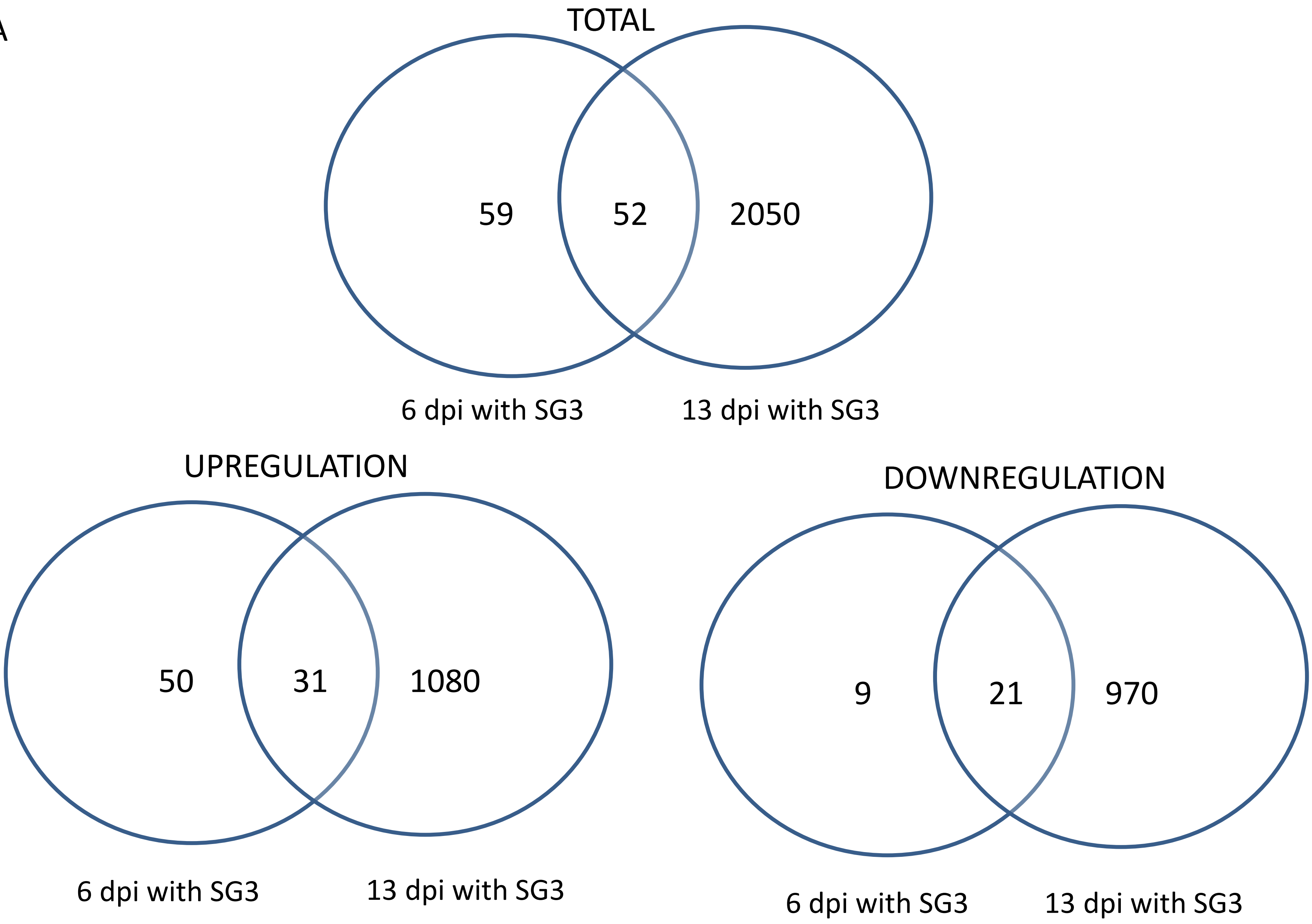

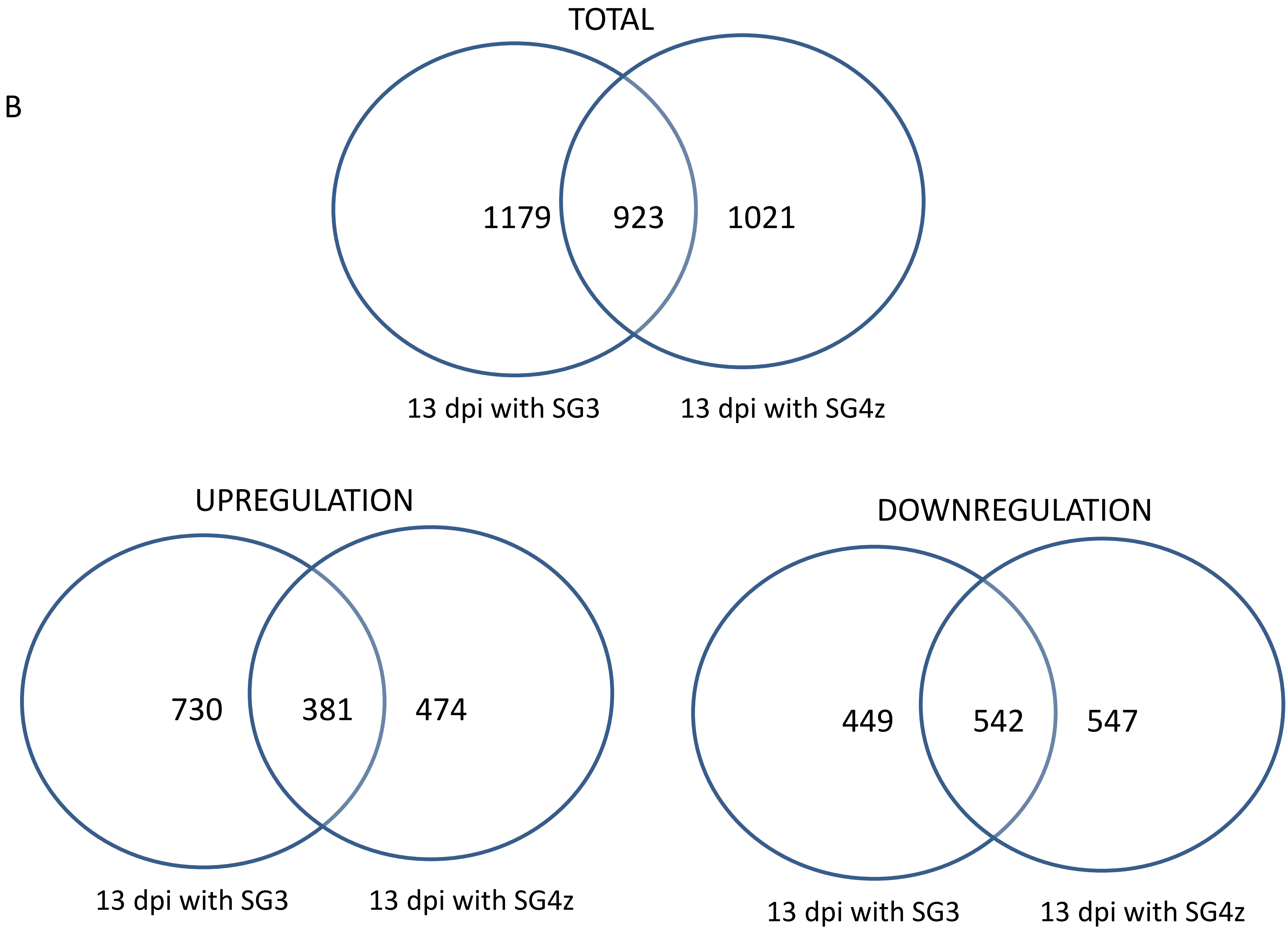
B
TOTAL
1179
923
1021
13 dpi with SG3
13 dpi with SG4z
UPREGULATION
730
381
474
13 dpi with SG3
13 dpi with SG4z
DOWNREGULATION
449
542
547
13 dpi with SG3
13 dpi with SG4z
