# Supplementary material for: Global changes in gene expression during compatible and incompatible interactions of cowpea (Vigna unguiculata L.) with the root parasitic angiosperm Striga gesnerioides
Source: BMC Genomics. 2012 Aug 17;13:402. doi: 10.1186/1471-2164-13-402 (PMC3505475; doi:10.1186/1471-2164-13-402)
Supplement: Additional file 4 — Quantitative reverse-transcriptase PCR verification of differential gene expression. [file 1471-2164-13-402-S4.pdf]

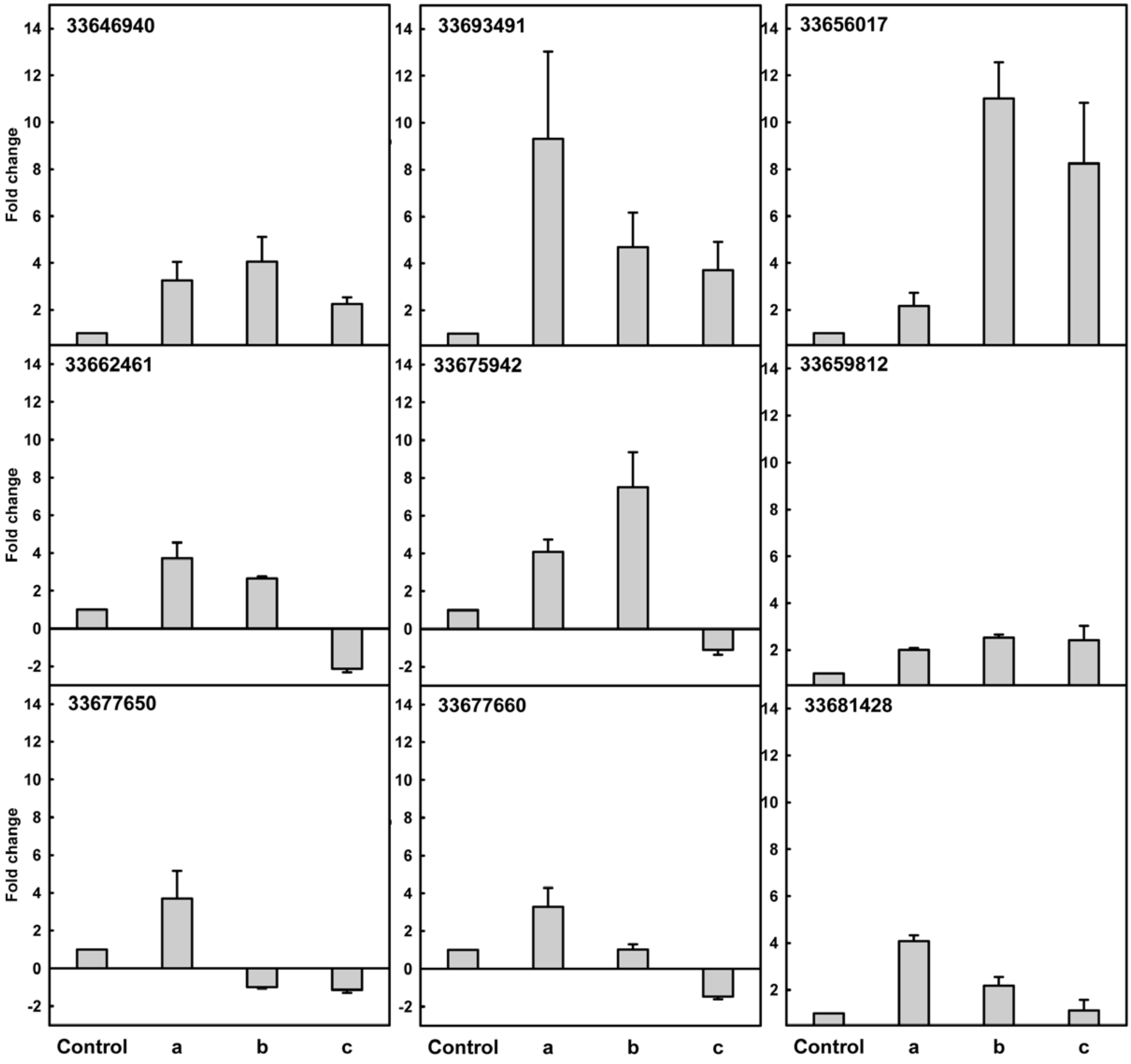

Additional file 4 Quantitative reverse-transcriptase PCR verification of differential gene expression

Data presented are fold-change values relative to control sample in (a) Cowpea cultivar infected with S. gesnerioides race SG3 at 6 dpi (b) cowpea cultivar B301 infected with S. gesnerioides race SG3 at 13 dpi (c) cowpea cultivar B301 infected with *S. gesnerioides* race SG4z at 13 dpi.
