# Supplementary figures and images for: Global changes in gene expression during compatible and incompatible interactions of cowpea (Vigna unguiculata L.) with the root parasitic angiosperm Striga gesnerioides
Source: BMC Genomics. 2012 Aug 17;13:402. doi: 10.1186/1471-2164-13-402 (PMC3505475; doi:10.1186/1471-2164-13-402)

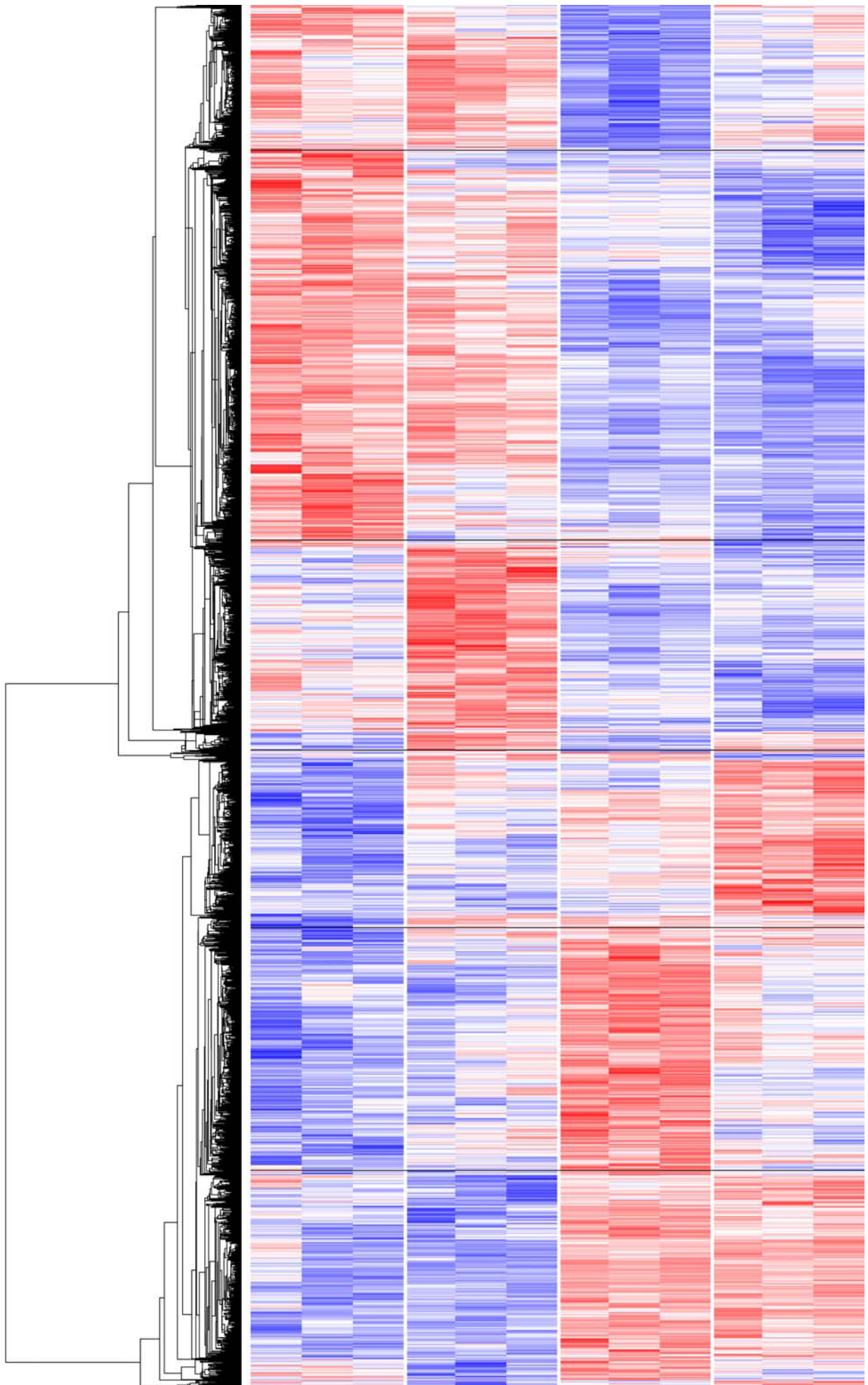

Additional file 5 Heat map 5%FDR

Supplement: Additional file 5 — Heat map 5% FDR. [file 1471-2164-13-402-S5.pdf]
