# Supplementary material for: Global changes in gene expression during compatible and incompatible interactions of cowpea (Vigna unguiculata L.) with the root parasitic angiosperm Striga gesnerioides
Source: BMC Genomics. 2012 Aug 17;13:402. doi: 10.1186/1471-2164-13-402 (PMC3505475; doi:10.1186/1471-2164-13-402)
Supplement: Additional file 6 — Representative genes from the heatmap showing a full expression profile at 0.1% FDR threshold. [file 1471-2164-13-402-S6.pdf]

Cowpea-Striga Virulence Susceptibility Study

Shom Paul and Aaron Mackey

March 16, 2012

This catalog contains selected genes of interest.

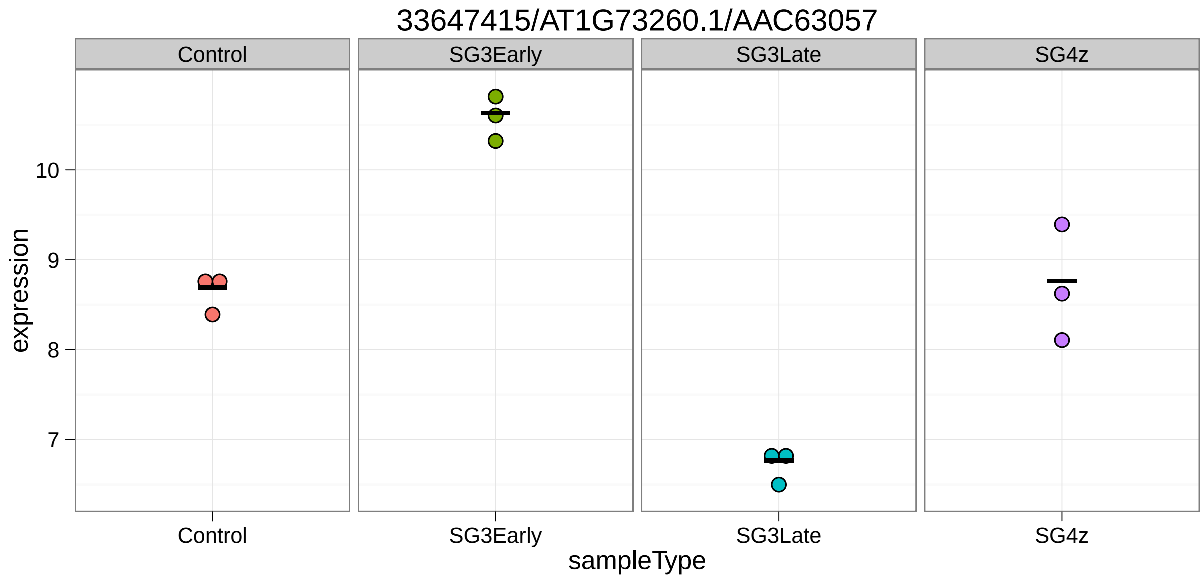

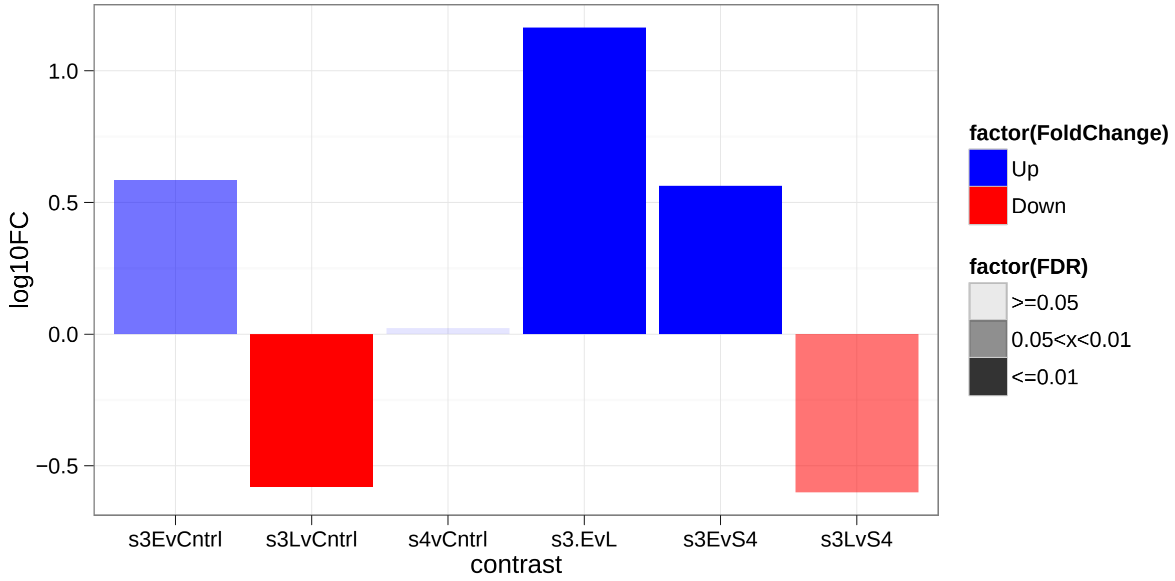

	contrast	logFC	adjPVal
1	s3EvCntrl	1.9	0.023
2	s3LvCntrl	-1.9	0.0041
3	s4vCntrl	0.071	0.94
4	s3.EvL	3.9	0.00017
5	s3EvS4	1.9	0.0049
6	s3LvS4	-2	0.027

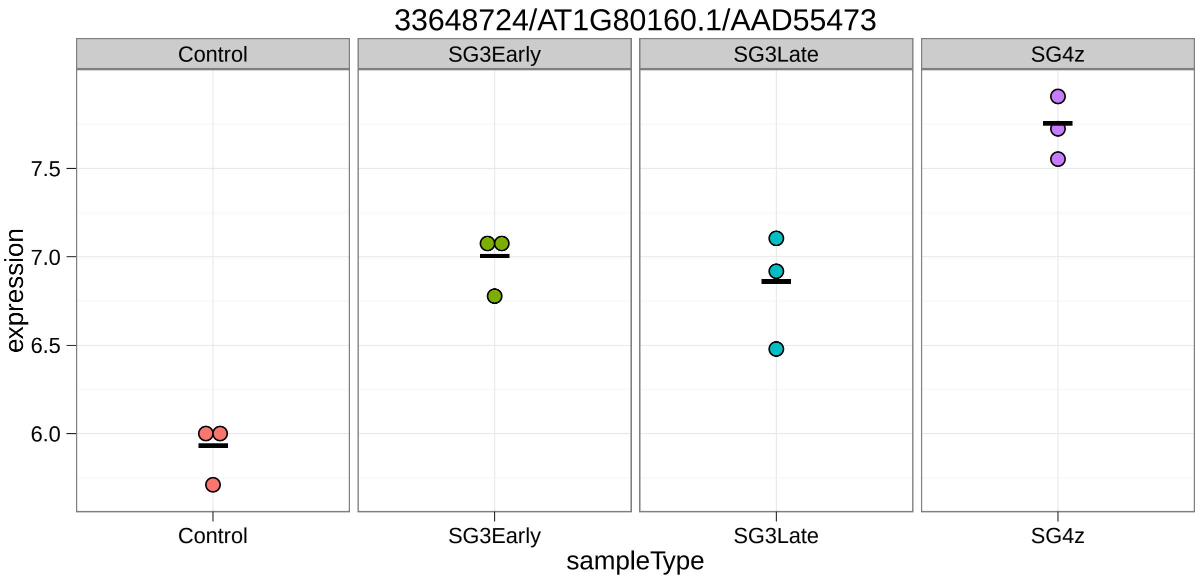

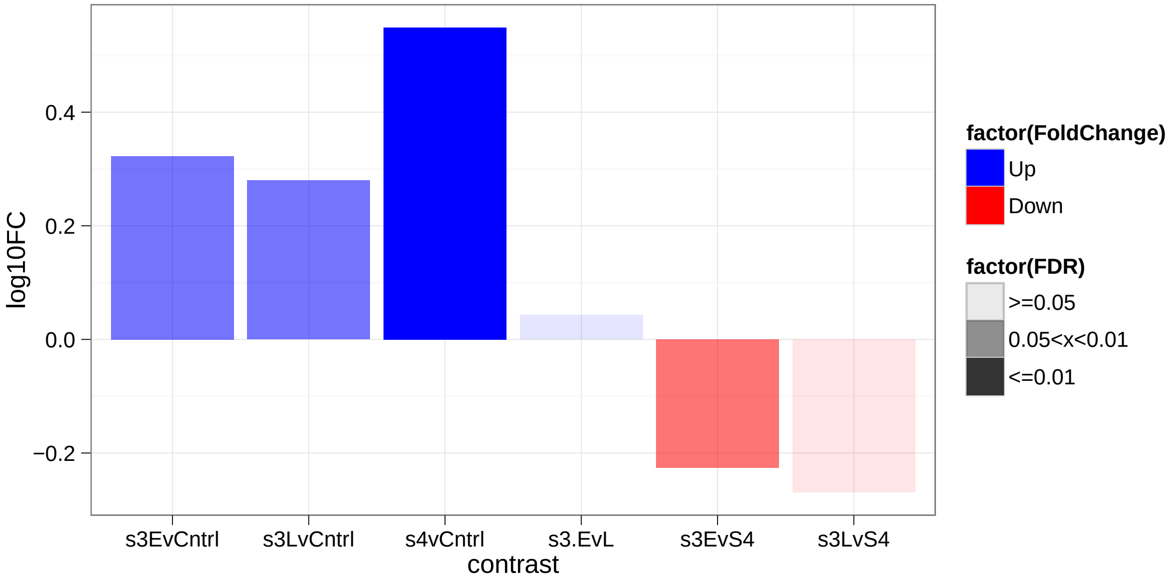

	contrast	logFC	adjPVal
1	s3EvCntrl	1.1	0.04
2	s3LvCntrl	0.93	0.015
3	s4vCntrl	1.8	0.00042
4	s3.EvL	0.14	0.8
5	s3EvS4	-0.75	0.042
6	s3LvS4	-0.89	0.084

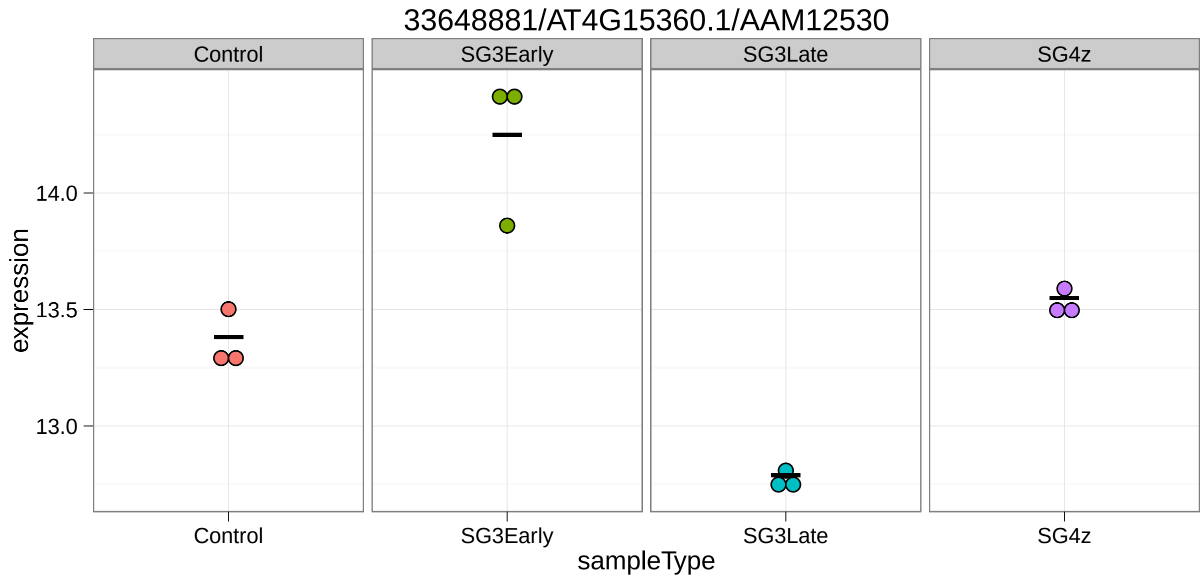

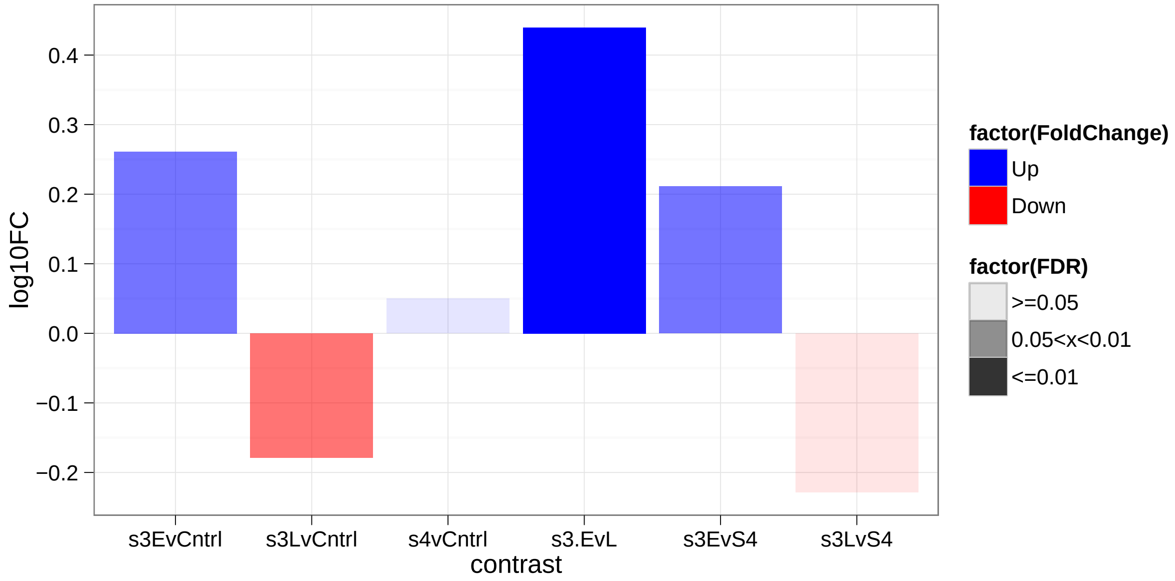

	contrast	logFC	adjPVal
1	s3EvCntrl	0.87	0.05
2	s3LvCntrl	-0.59	0.05
3	s4vCntrl	0.17	0.66
4	s3.EvL	1.5	0.00094
5	s3EvS4	0.7	0.028
6	s3LvS4	-0.76	0.084

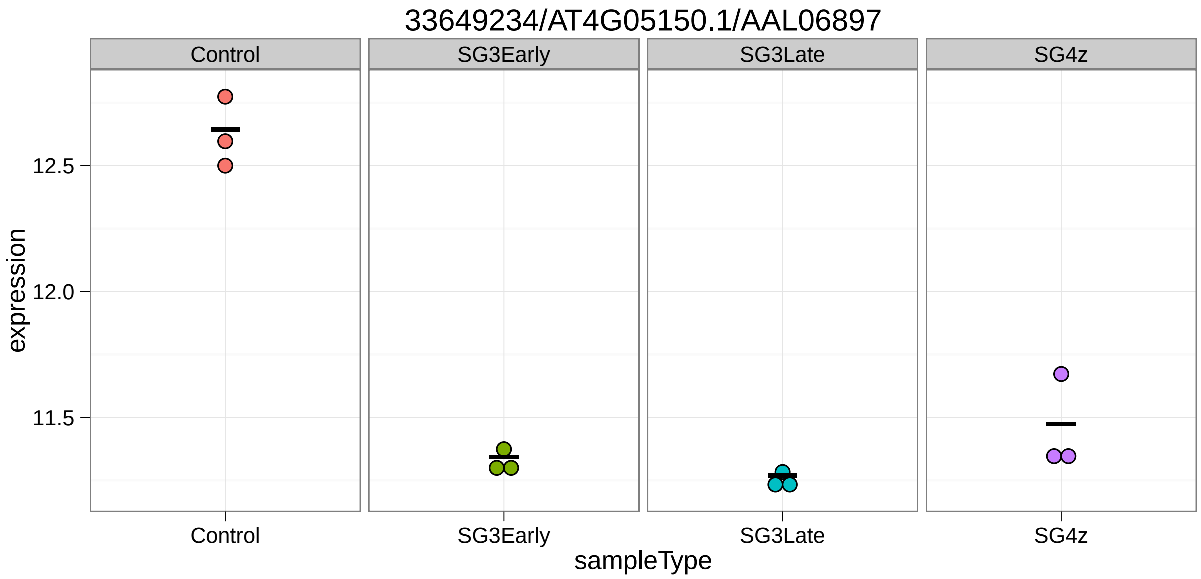

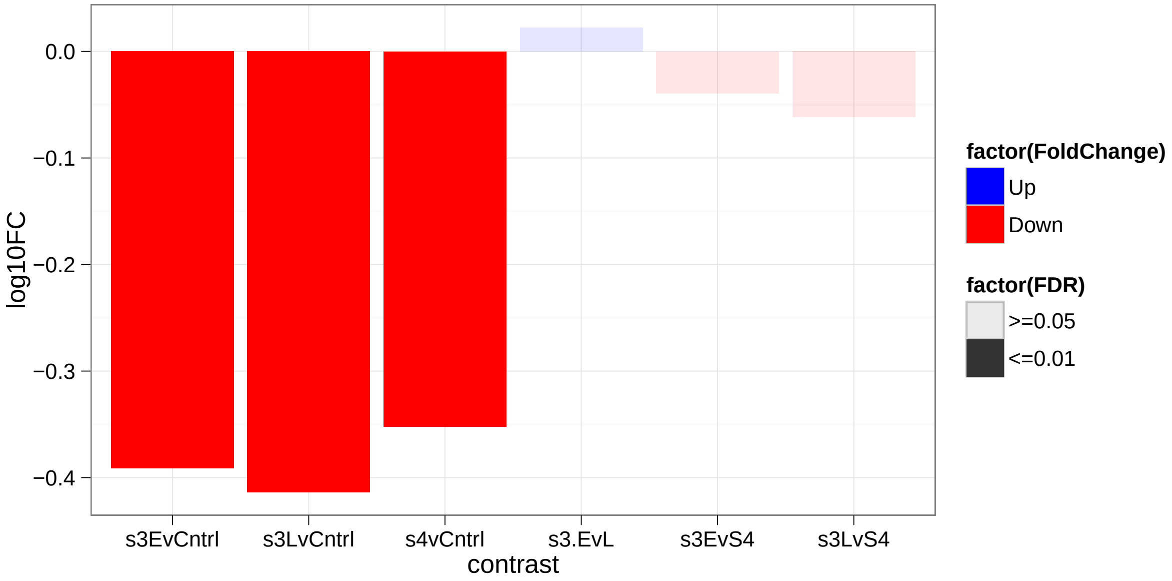

	contrast	logFC	adjPVal
1	s3EvCntrl	-1.3	0.0017
2	s3LvCntrl	-1.4	0.00041
3	s4vCntrl	-1.2	0.00057
4	s3.EvL	0.074	0.86
5	s3EvS4	-0.13	0.71
6	s3LvS4	-0.21	0.65

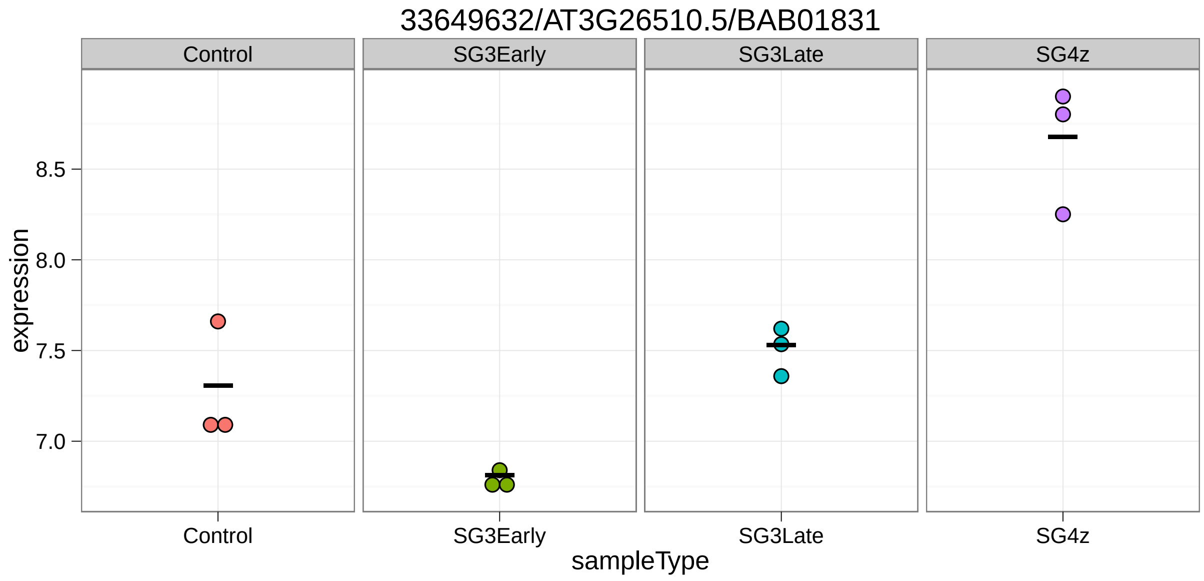

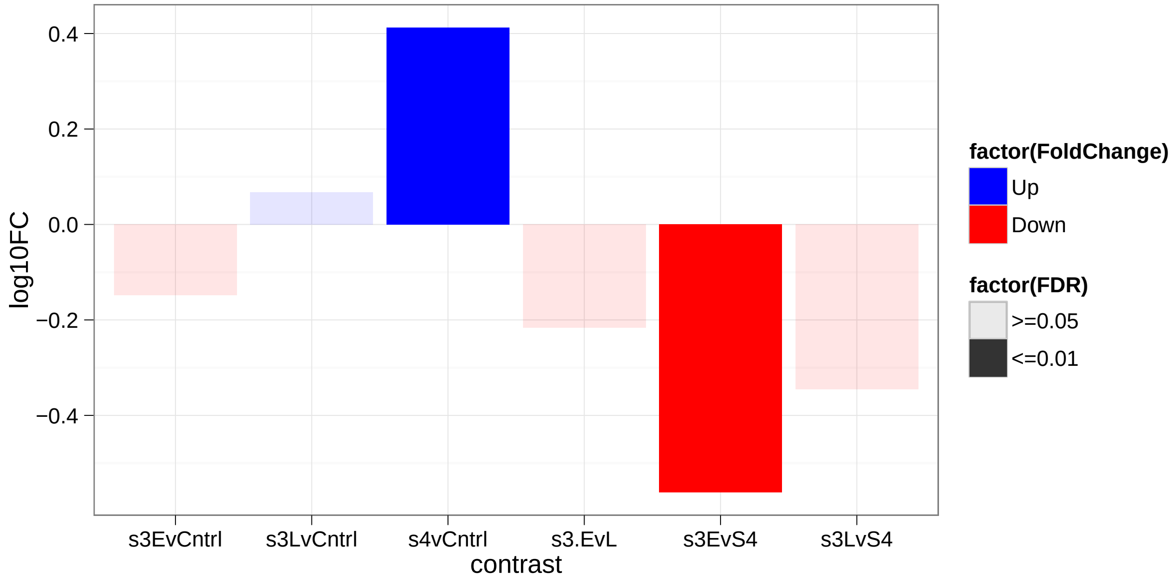

	contrast	logFC	adjPVal
1	s3EvCntrl	-0.49	0.37
2	s3LvCntrl	0.22	0.62
3	s4vCntrl	1.4	0.0032
4	s3.EvL	-0.72	0.073
5	s3EvS4	-1.9	0.0009
6	s3LvS4	-1.1	0.055

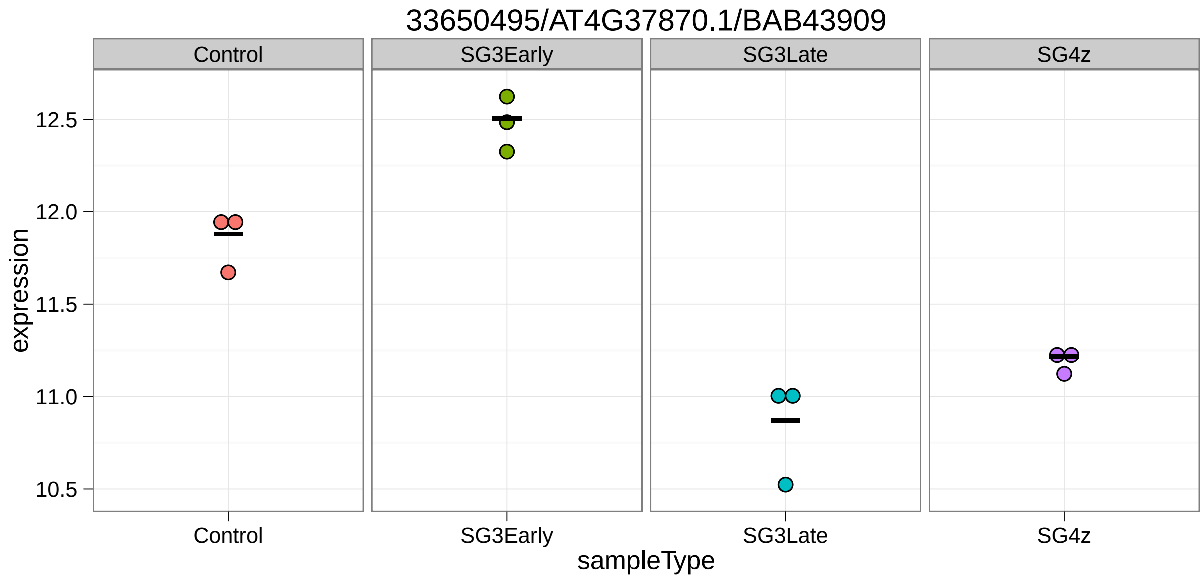

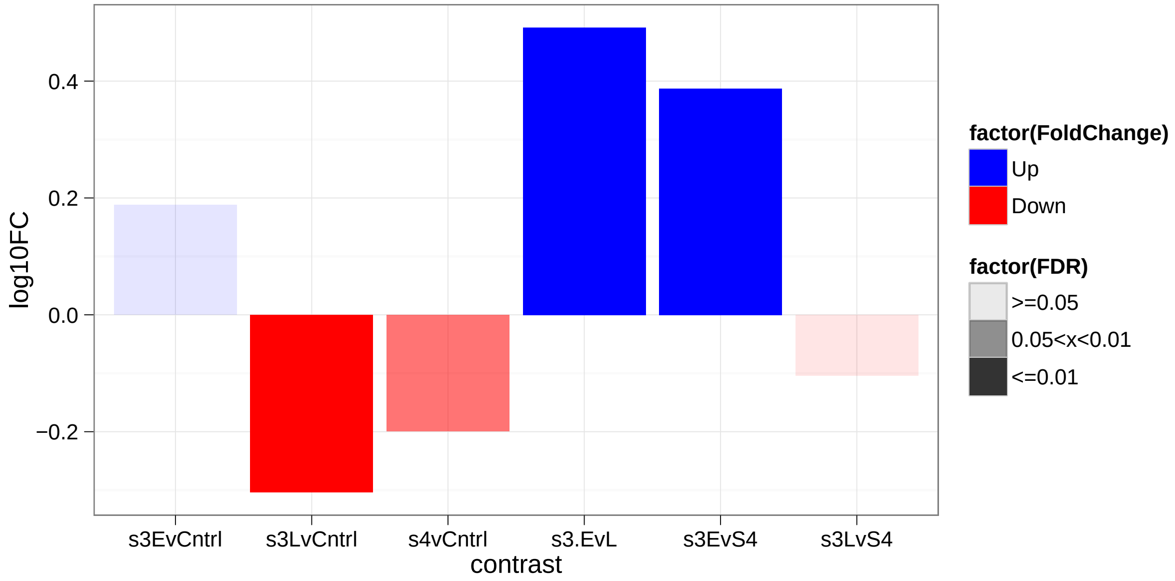

	contrast	logFC	adjPVal
1	s3EvCntrl	0.62	0.15
2	s3LvCntrl	-1	0.0051
3	s4vCntrl	-0.66	0.035
4	s3.EvL	1.6	0.00059
5	s3EvS4	1.3	0.0018
6	s3LvS4	-0.35	0.48

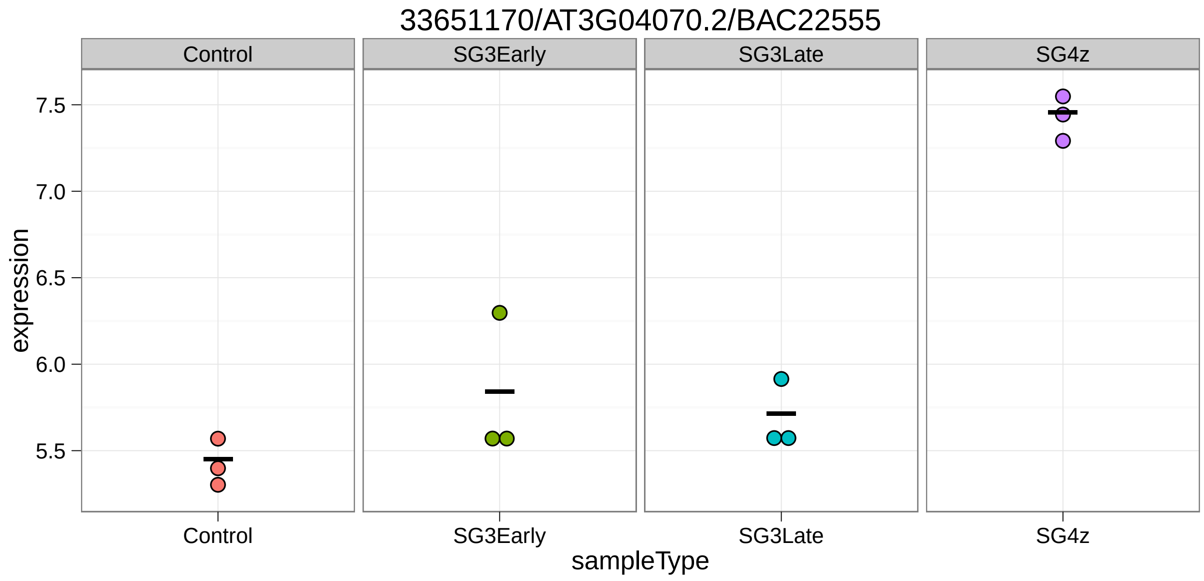

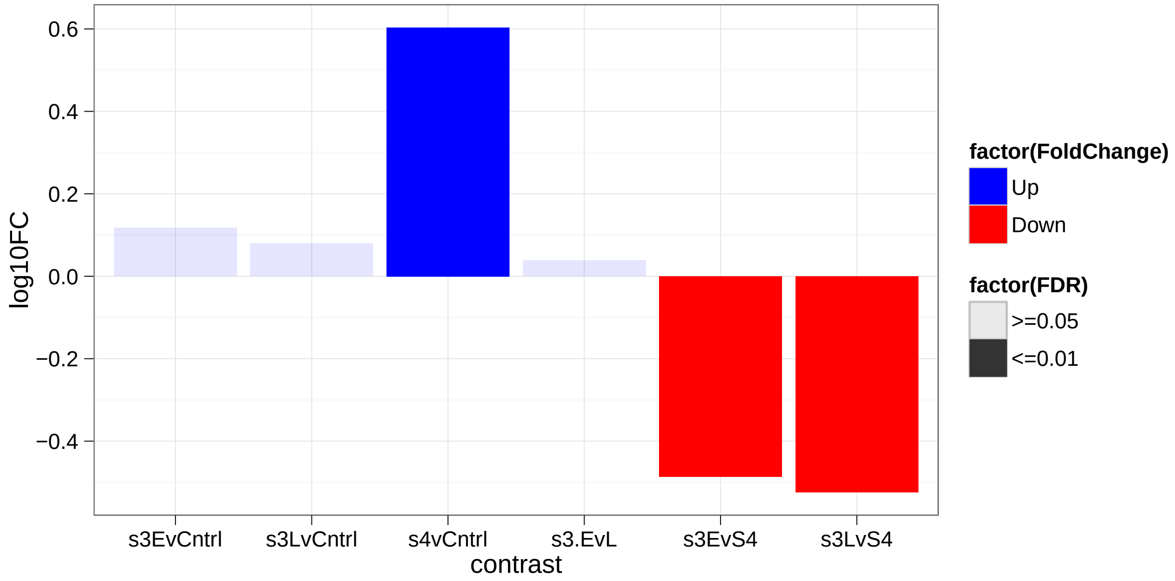

	contrast	logFC	adjPVal
1	s3EvCntrl	0.39	0.48
2	s3LvCntrl	0.26	0.54
3	s4vCntrl	2	0.00044
4	s3.EvL	0.13	0.85
5	s3EvS4	-1.6	0.002
6	s3LvS4	-1.7	0.0096

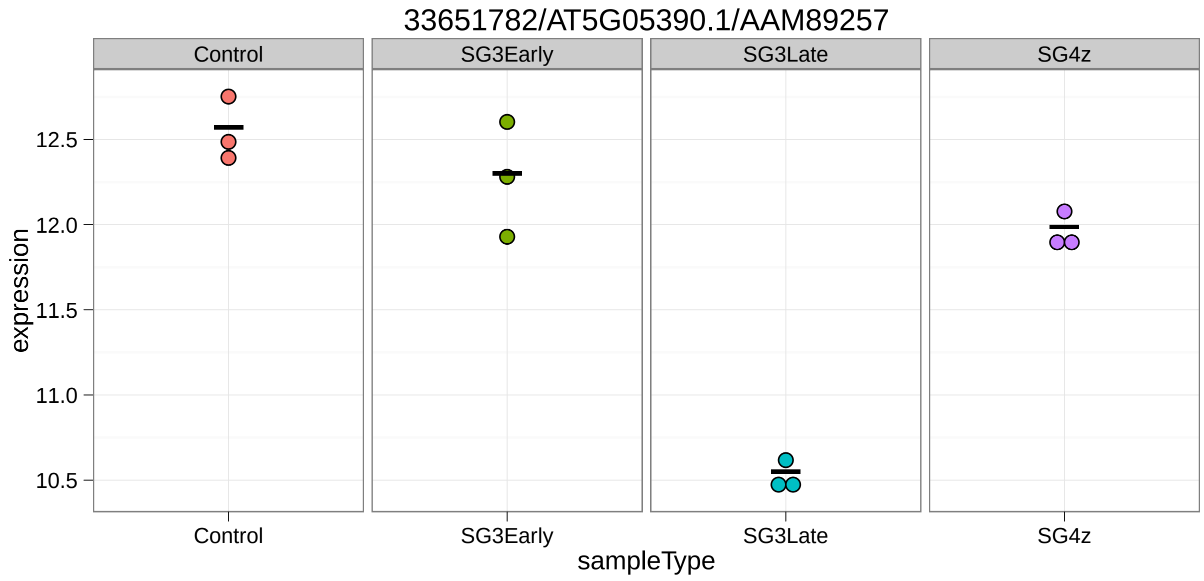

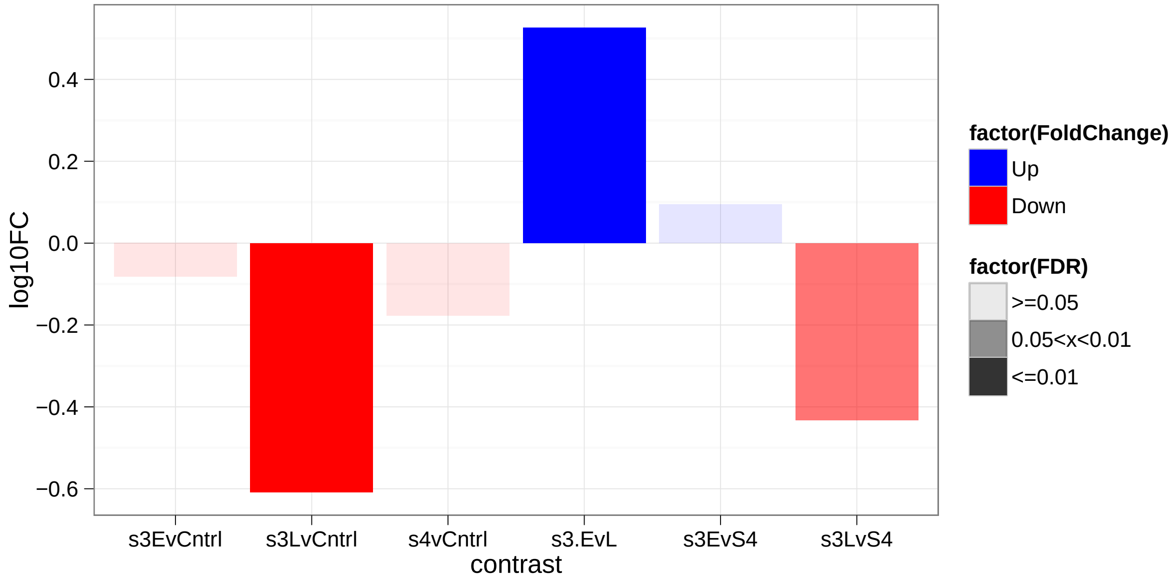

	contrast	logFC	adjPVal
1	s3EvCntrl	-0.27	0.58
2	s3LvCntrl	-2	0.00036
3	s4vCntrl	-0.59	0.085
4	s3.EvL	1.7	0.00067
5	s3EvS4	0.31	0.43
6	s3LvS4	-1.4	0.011

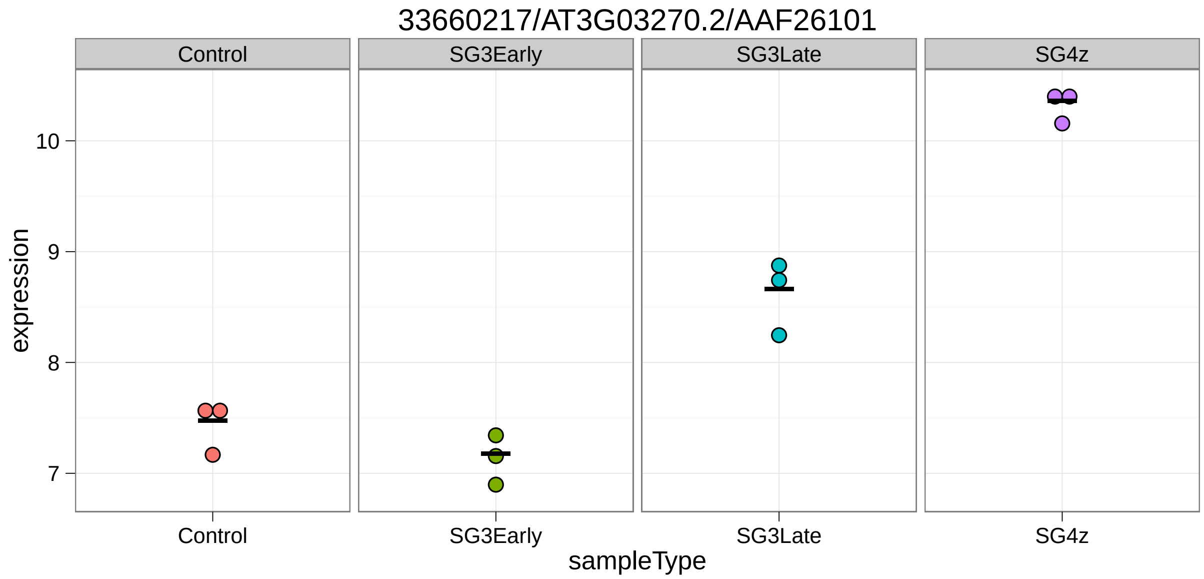

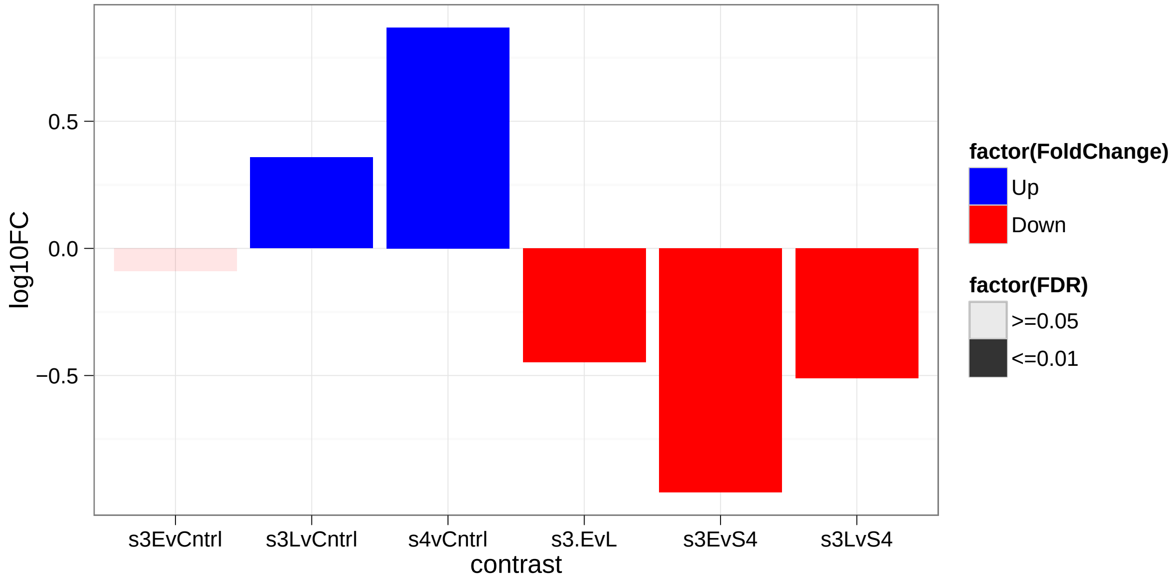

	contrast	logFC	adjPVal
1	s3EvCntrl	-0.3	0.59
2	s3LvCntrl	1.2	0.0066
3	s4vCntrl	2.9	5.7e-05
4	s3.EvL	-1.5	0.0026
5	s3EvS4	-3.2	6.4e-05
6	s3LvS4	-1.7	0.0096

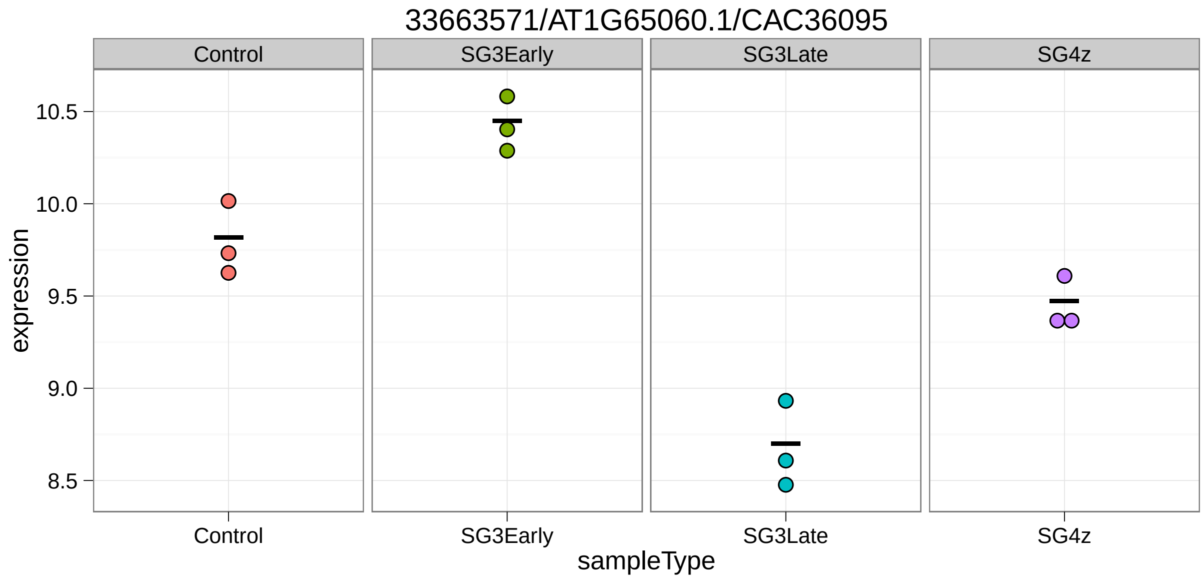

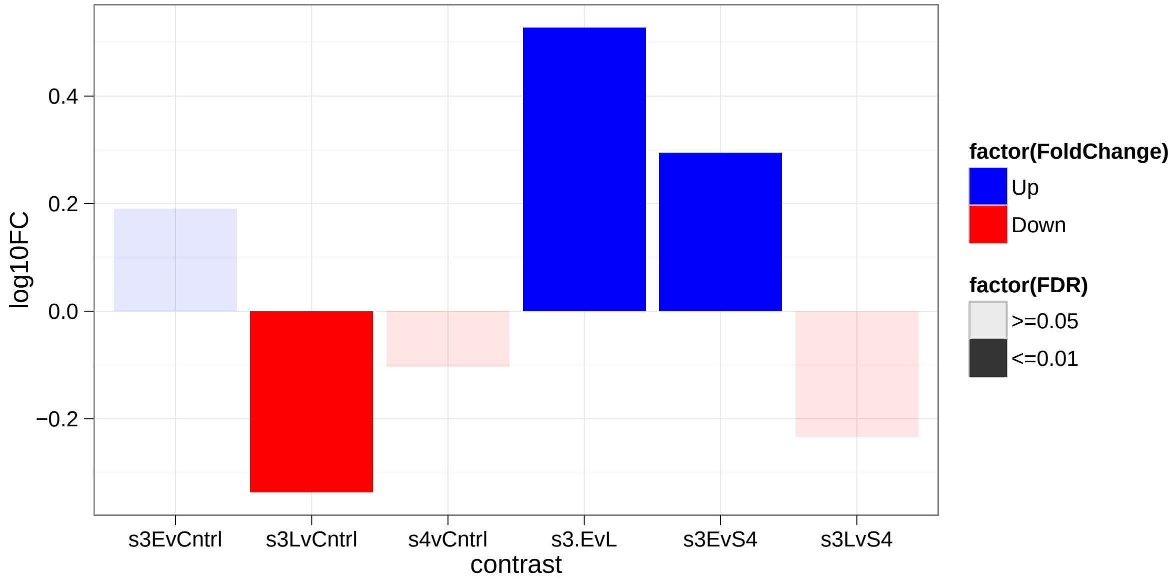

	contrast	logFC	adjPVal
1	s3EvCntrl	0.63	0.15
2	s3LvCntrl	-1.1	0.0034
3	s4vCntrl	-0.34	0.31
4	s3.EvL	1.8	0.00043
5	s3EvS4	0.98	0.0071
6	s3LvS4	-0.78	0.089

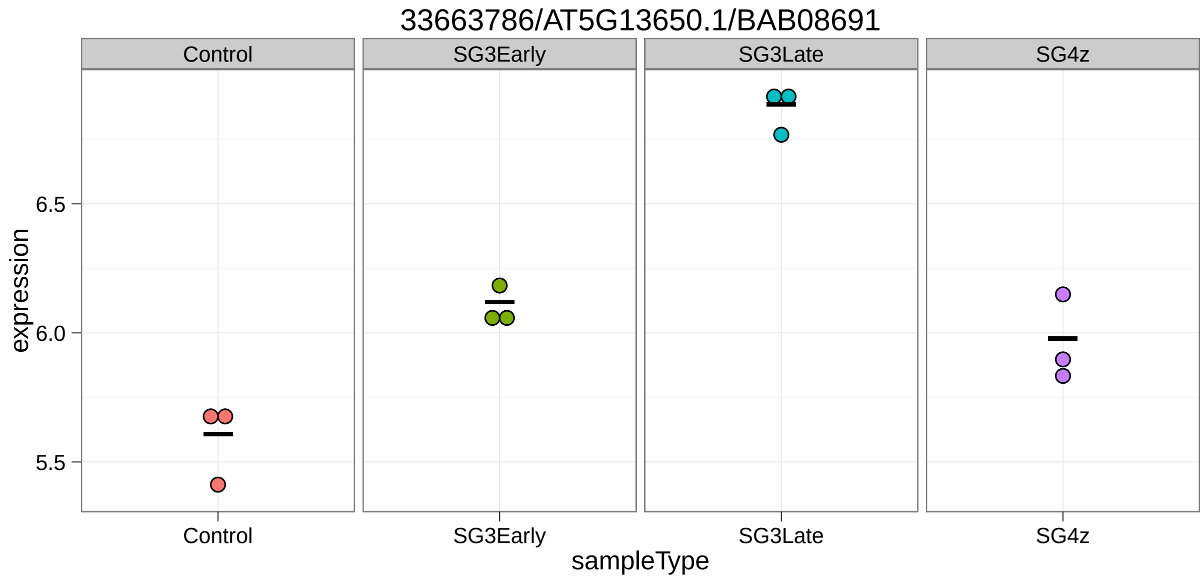

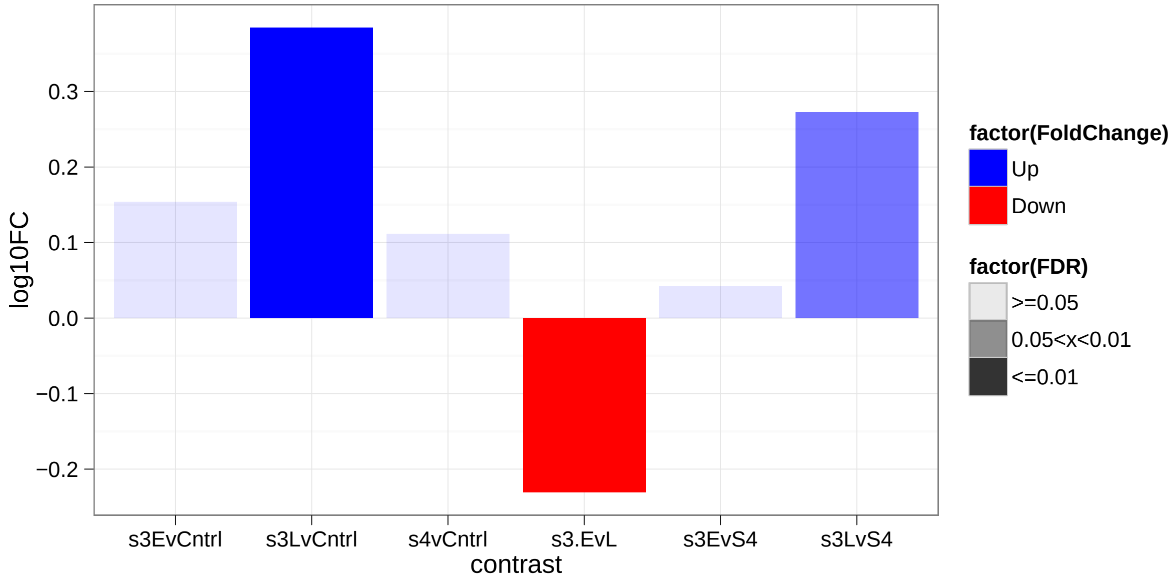

	contrast	logFC	adjPVal
1	s3EvCntrl	0.51	0.14
2	s3LvCntrl	1.3	0.00063
3	s4vCntrl	0.37	0.14
4	s3.EvL	-0.77	0.0073
5	s3EvS4	0.14	0.69
6	s3LvS4	0.91	0.025

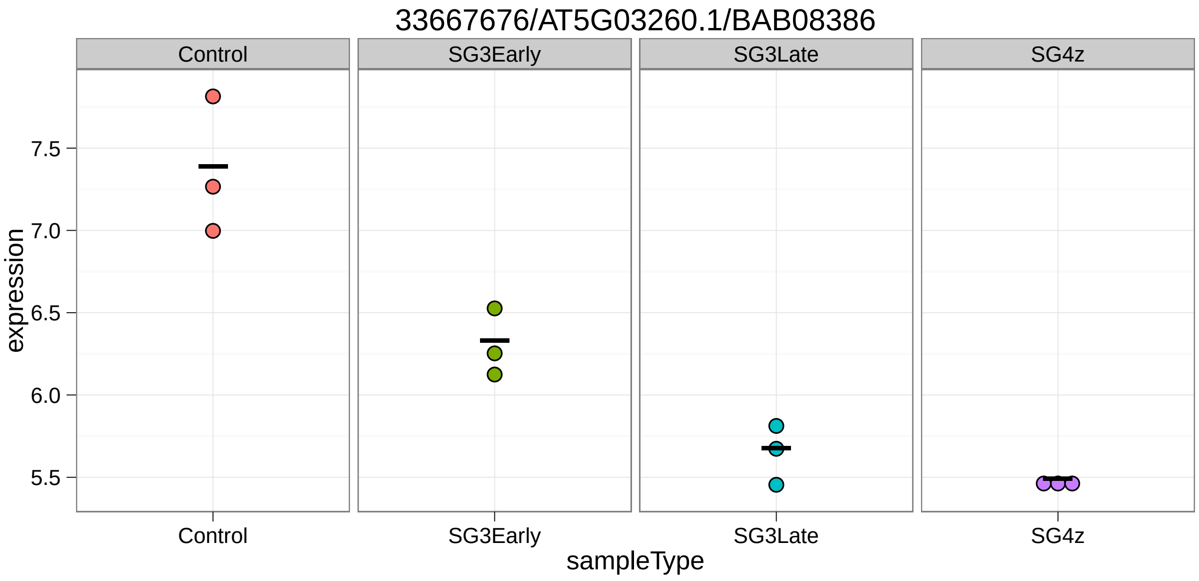

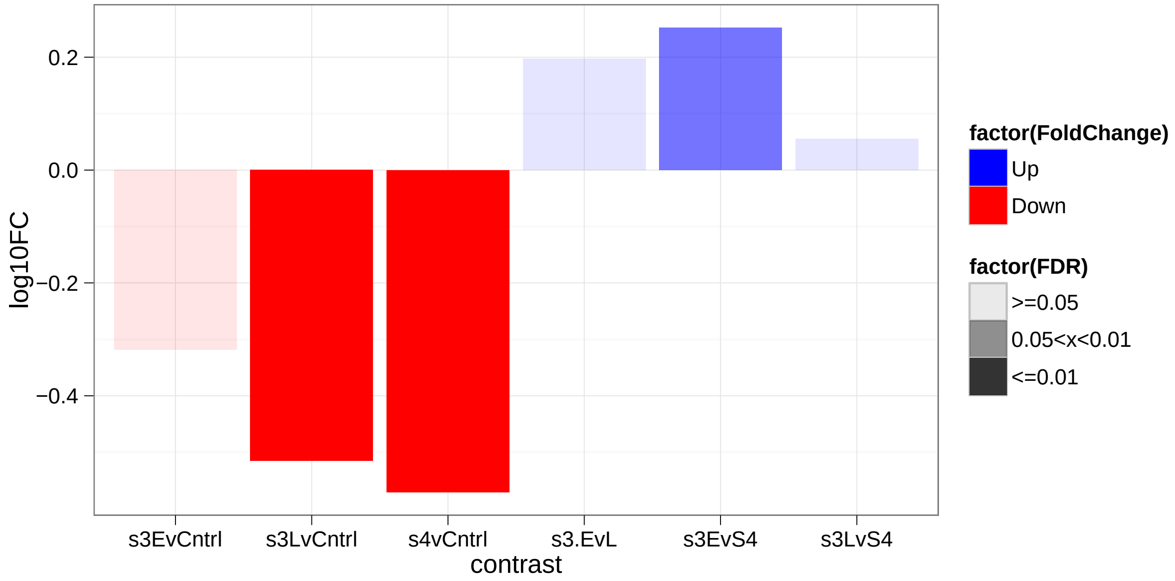

	contrast	logFC	adjPVal
1	s3EvCntrl	-1.1	0.066
2	s3LvCntrl	-1.7	0.0012
3	s4vCntrl	-1.9	0.0006
4	s3.EvL	0.65	0.098
5	s3EvS4	0.84	0.04
6	s3LvS4	0.18	0.86

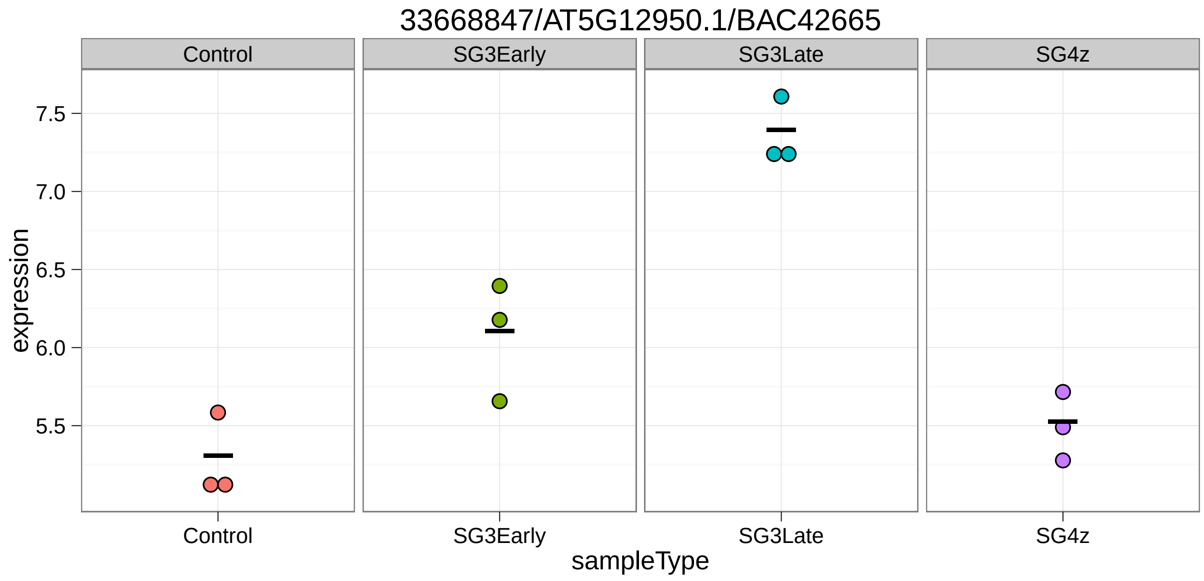

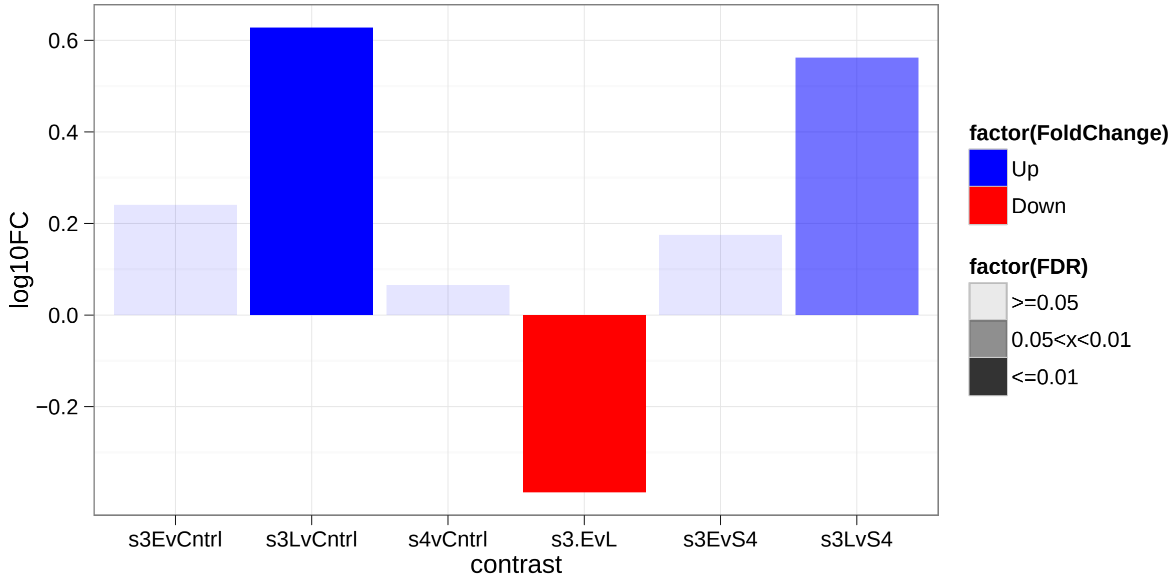

	contrast	logFC	adjPVal
1	s3EvCntrl	0.8	0.19
2	s3LvCntrl	2.1	0.00082
3	s4vCntrl	0.22	0.7
4	s3.EvL	-1.3	0.0087
5	s3EvS4	0.58	0.2
6	s3LvS4	1.9	0.011

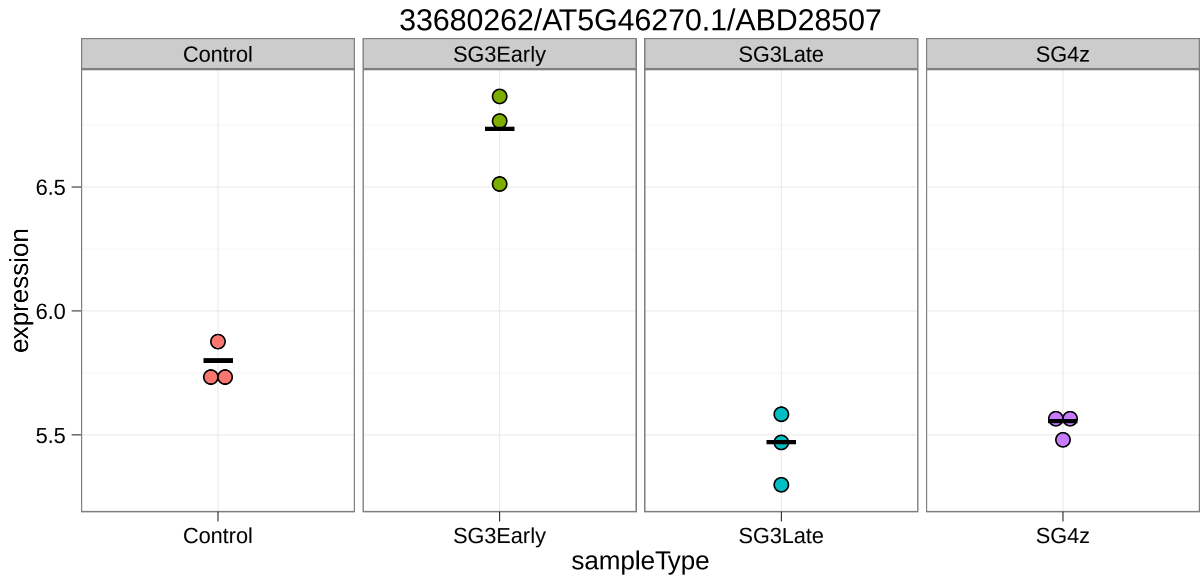

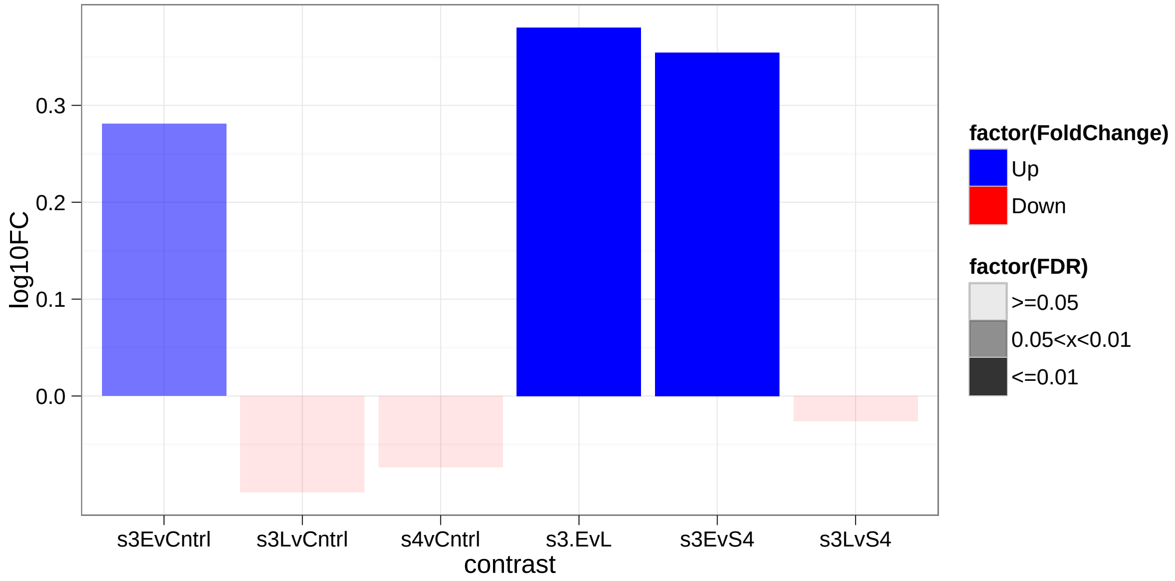

	contrast	logFC	adjPVal
1	s3EvCntrl	0.93	0.015
2	s3LvCntrl	-0.33	0.17
3	s4vCntrl	-0.24	0.36
4	s3.EvL	1.3	0.00068
5	s3EvS4	1.2	0.00086
6	s3LvS4	-0.086	0.91

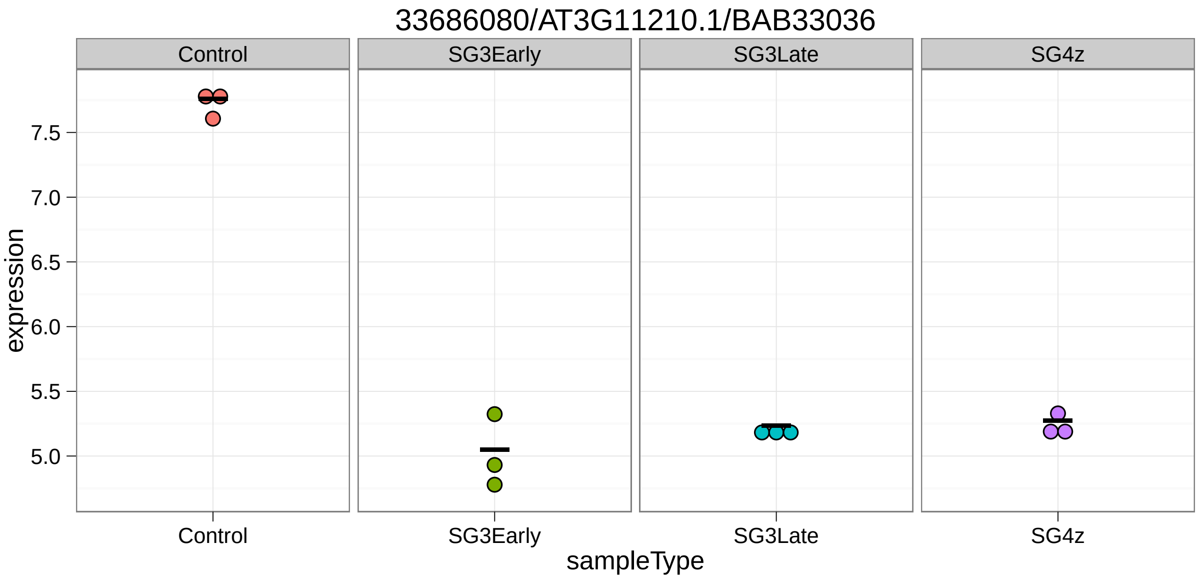

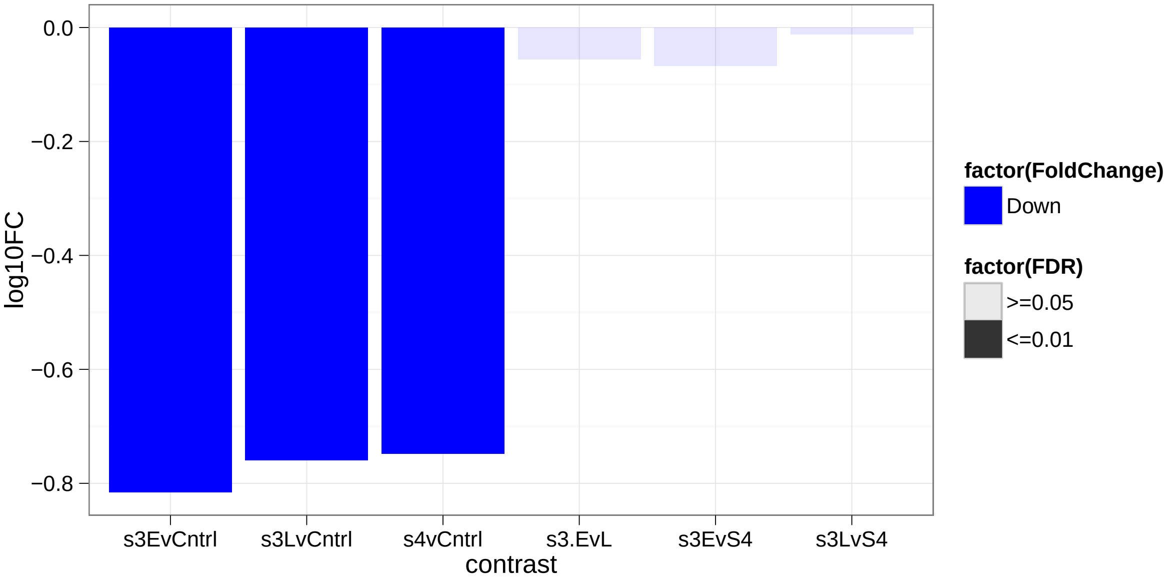

	contrast	logFC	adjPVal
1	s3EvCntrl	-2.7	1.3e-05
2	s3LvCntrl	-2.5	2.4e-05
3	s4vCntrl	-2.5	1.6e-05
4	s3.EvL	-0.19	0.59
5	s3EvS4	-0.22	0.52
6	s3LvS4	-0.038	0.98

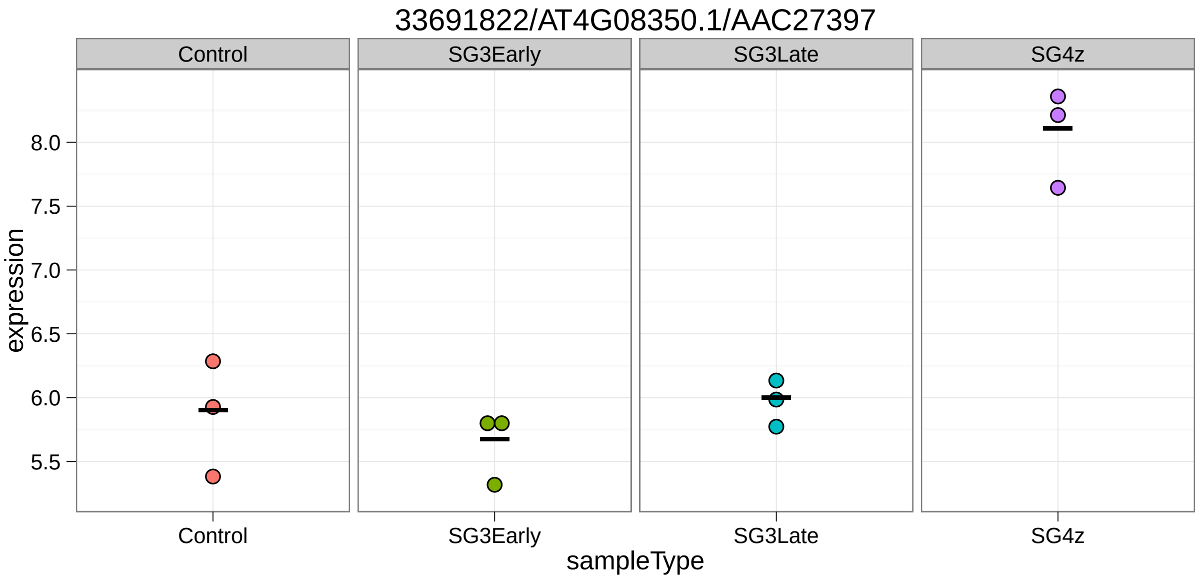

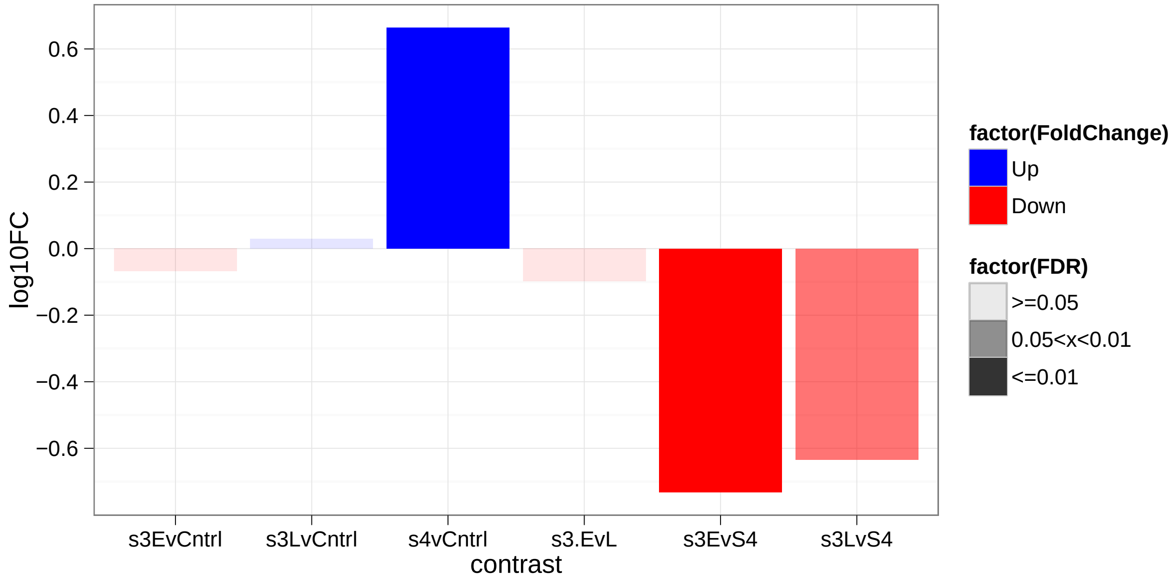

	contrast	logFC	adjPVal
1	s3EvCntrl	-0.23	0.78
2	s3LvCntrl	0.099	0.89
3	s4vCntrl	2.2	0.001
4	s3.EvL	-0.32	0.62
5	s3EvS4	-2.4	0.00086
6	s3LvS4	-2.1	0.012

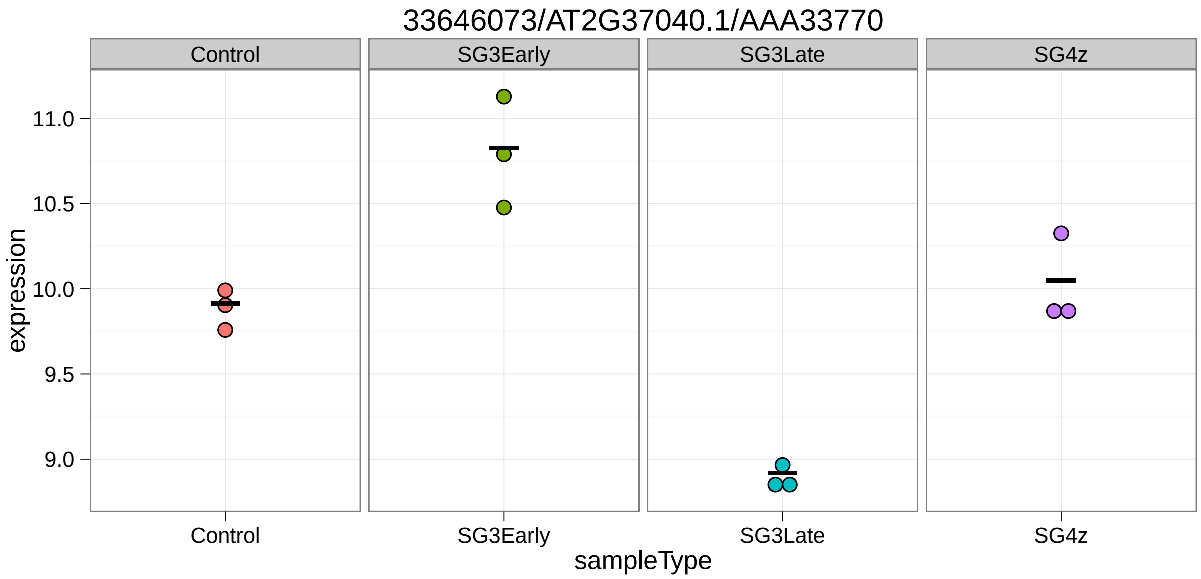

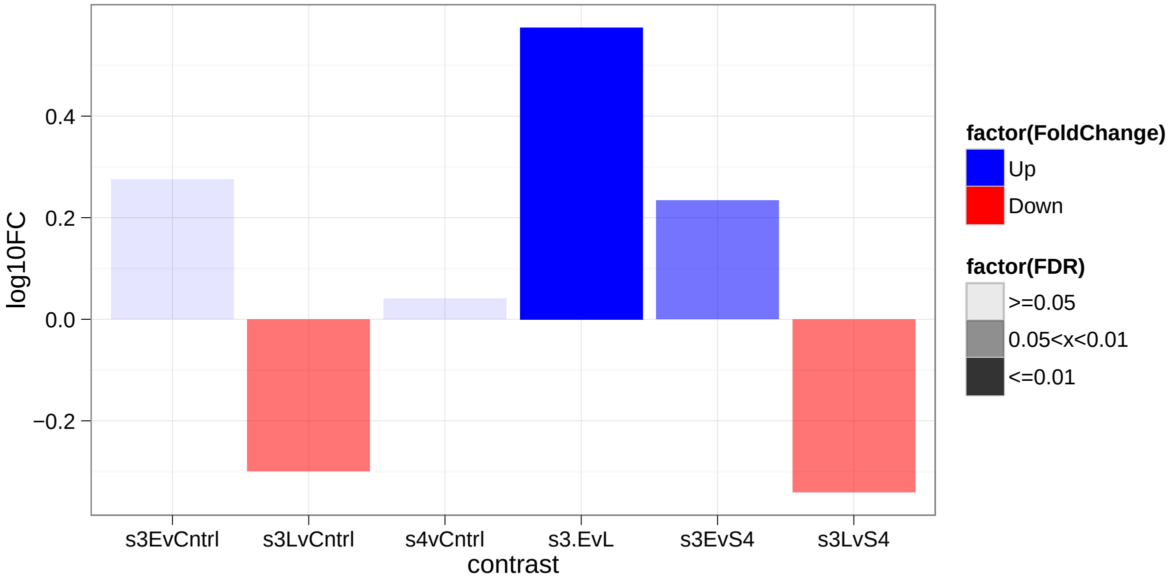

	contrast	logFC	adjPVal
1	s3EvCntrl	0.91	0.079
2	s3LvCntrl	-0.99	0.011
3	s4vCntrl	0.14	0.78
4	s3.EvL	1.9	0.00059
5	s3EvS4	0.78	0.037
6	s3LvS4	-1.1	0.042

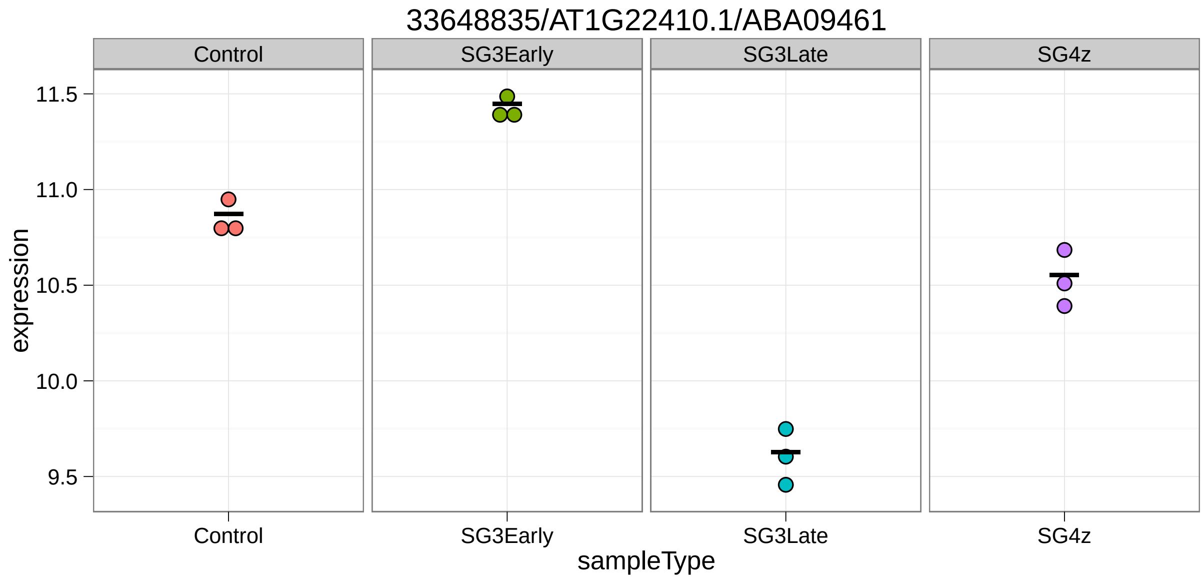

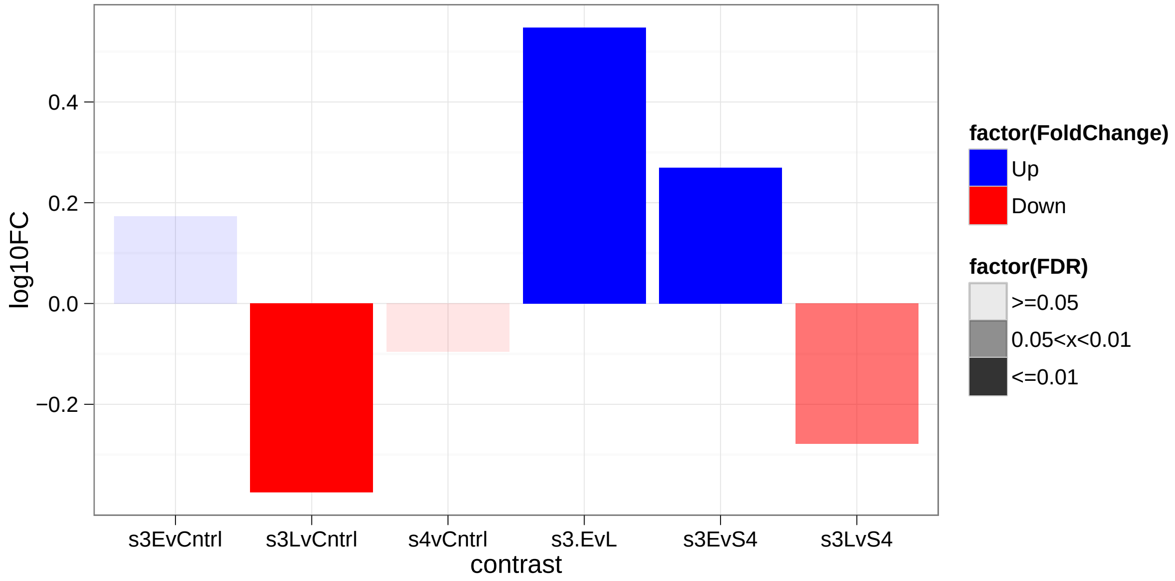

	contrast	logFC	adjPVal
1	s3EvCntrl	0.57	0.09
2	s3LvCntrl	-1.2	0.00059
3	s4vCntrl	-0.32	0.19
4	s3.EvL	1.8	0.00013
5	s3EvS4	0.89	0.0029
6	s3LvS4	-0.93	0.016

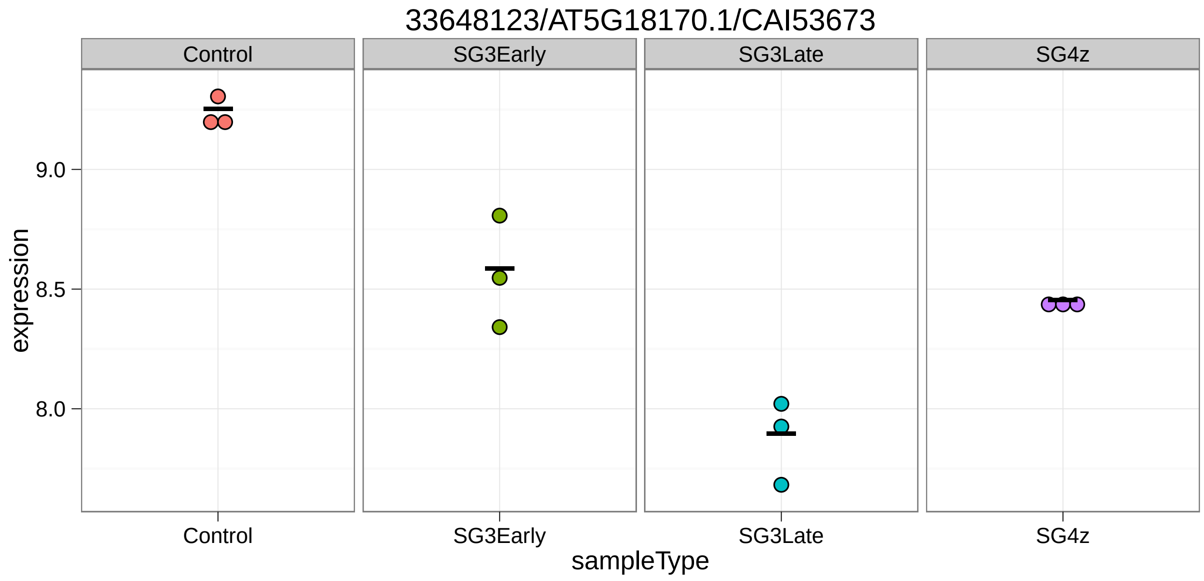

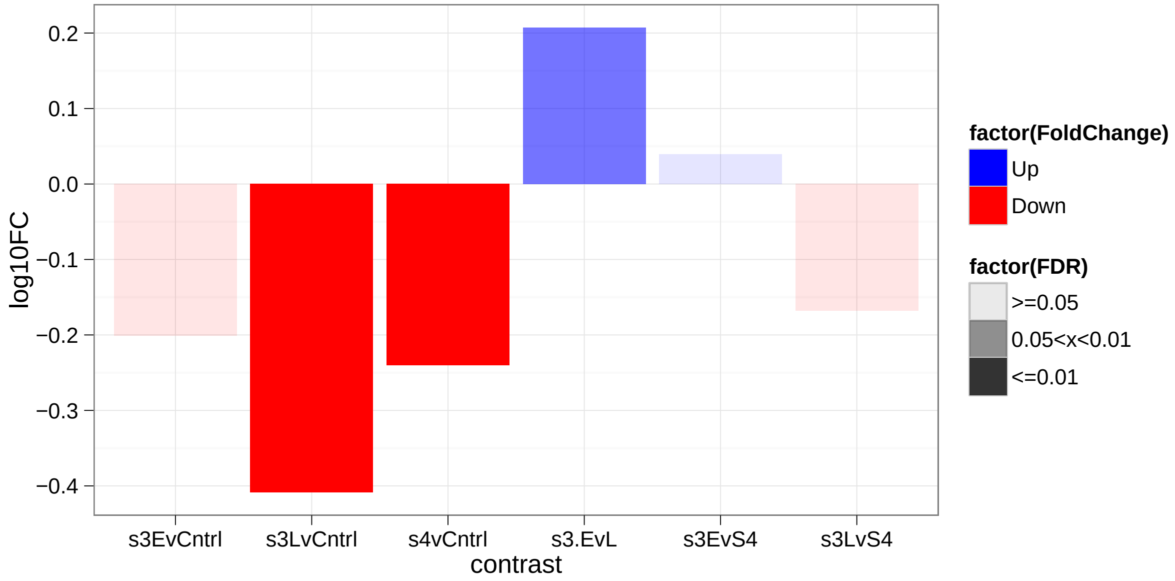

	contrast	logFC	adjPVal
1	s3EvCntrl	-0.67	0.087
2	s3LvCntrl	-1.4	0.0007
3	s4vCntrl	-0.8	0.0079
4	s3.EvL	0.69	0.018
5	s3EvS4	0.13	0.75
6	s3LvS4	-0.56	0.15

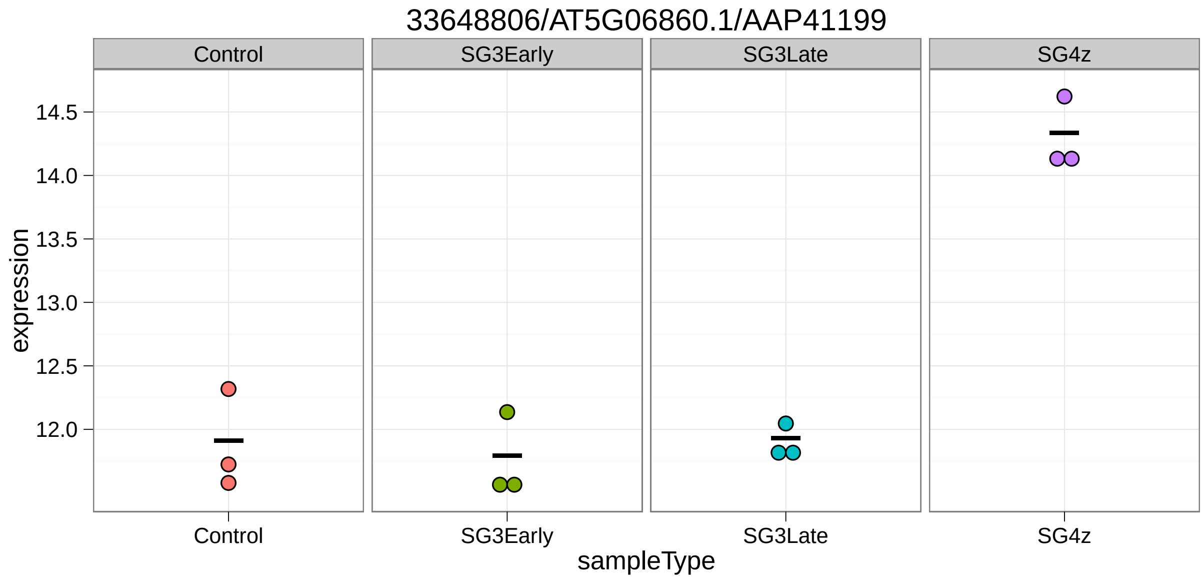

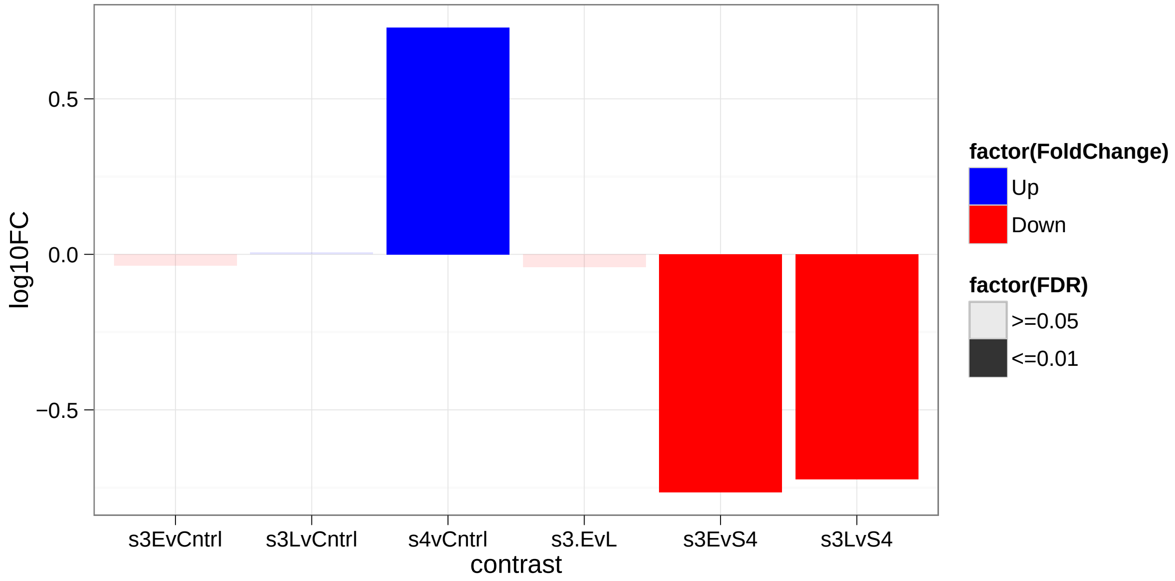

	contrast	logFC	adjPVal
1	s3EvCntrl	-0.12	0.88
2	s3LvCntrl	0.02	0.98
3	s4vCntrl	2.4	0.00038
4	s3.EvL	-0.14	0.86
5	s3EvS4	-2.5	0.00045
6	s3LvS4	-2.4	0.0045

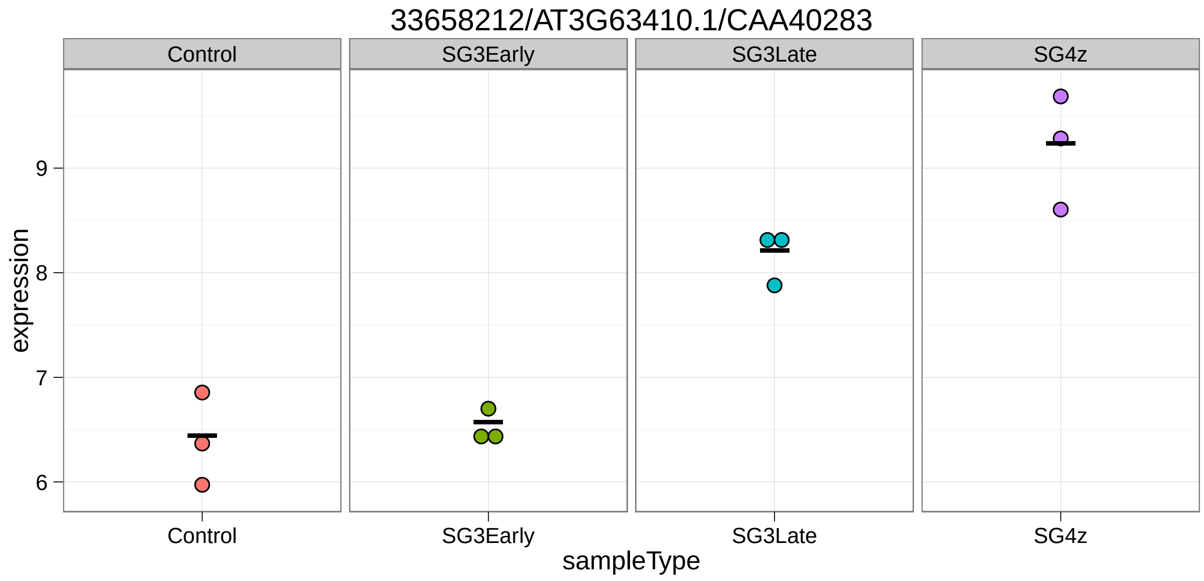

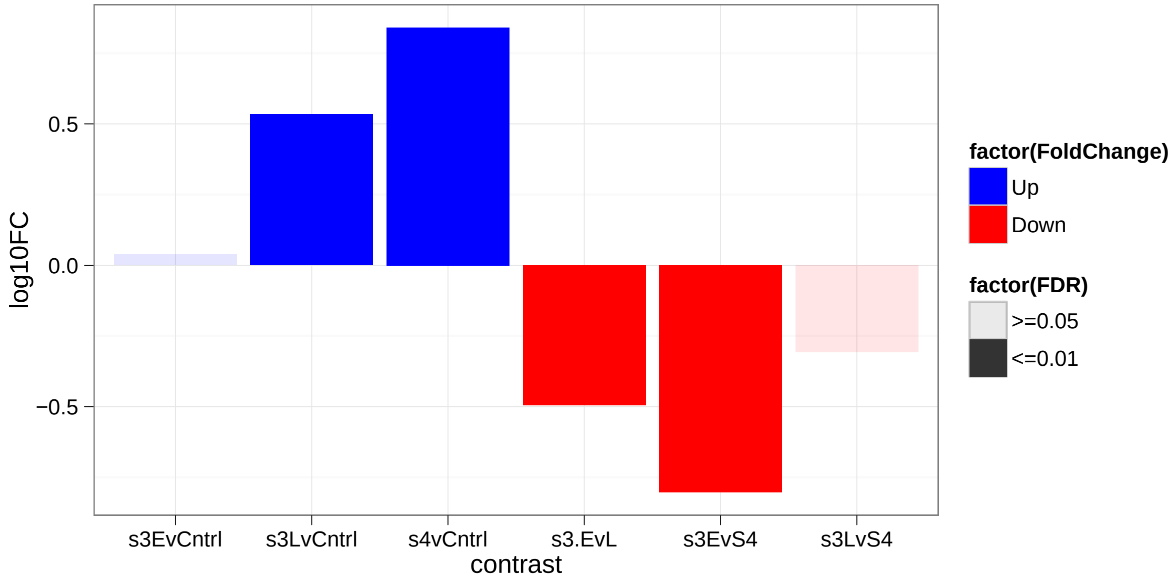

	contrast	logFC	adjPVal
1	s3EvCntrl	0.12	0.9
2	s3LvCntrl	1.8	0.0064
3	s4vCntrl	2.8	0.00056
4	s3.EvL	-1.6	0.0099
5	s3EvS4	-2.7	0.00093
6	s3LvS4	-1	0.21

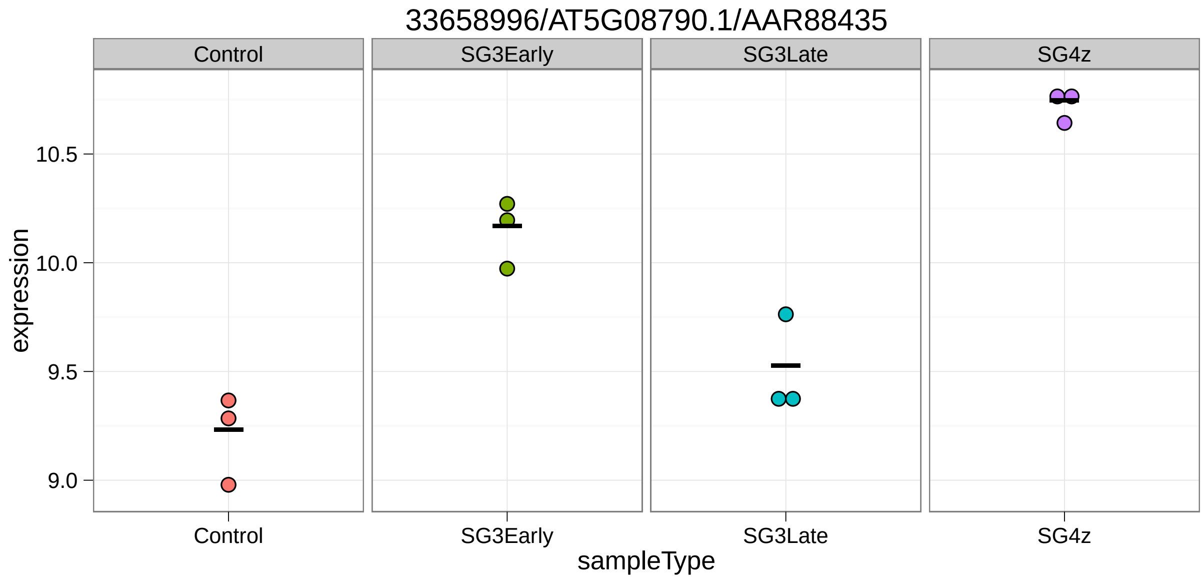

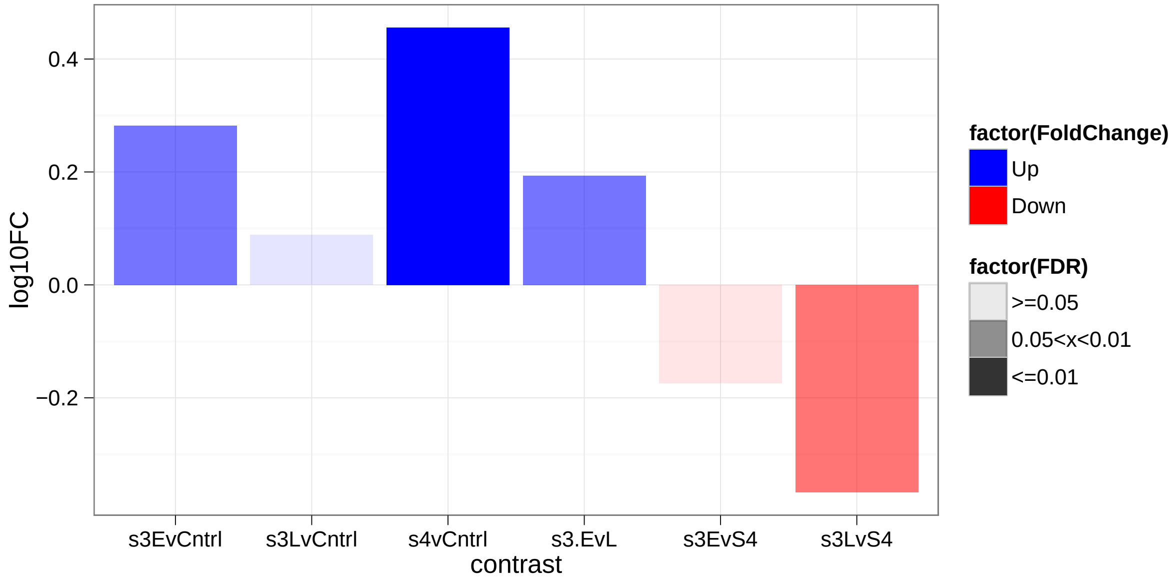

	contrast	logFC	adjPVal
1	s3EvCntrl	0.94	0.039
2	s3LvCntrl	0.29	0.33
3	s4vCntrl	1.5	0.00045
4	s3.EvL	0.64	0.038
5	s3EvS4	-0.58	0.062
6	s3LvS4	-1.2	0.012

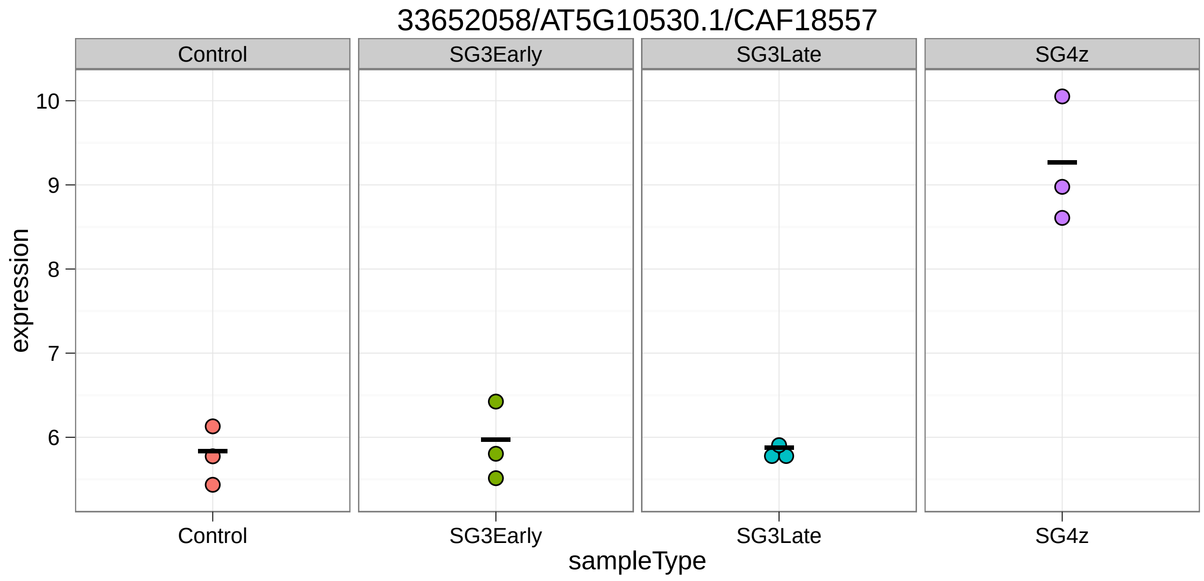

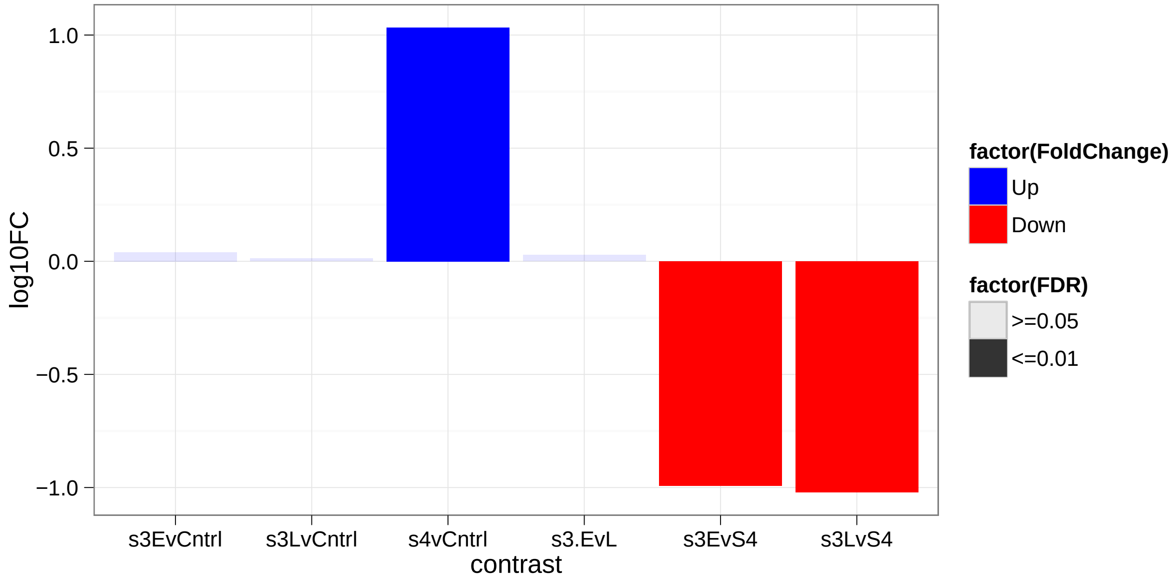

	contrast	logFC	adjPVal
1	s3EvCntrl	0.13	0.92
2	s3LvCntrl	0.041	0.97
3	s4vCntrl	3.4	0.00056
4	s3.EvL	0.094	0.95
5	s3EvS4	-3.3	0.00091
6	s3LvS4	-3.4	0.0054

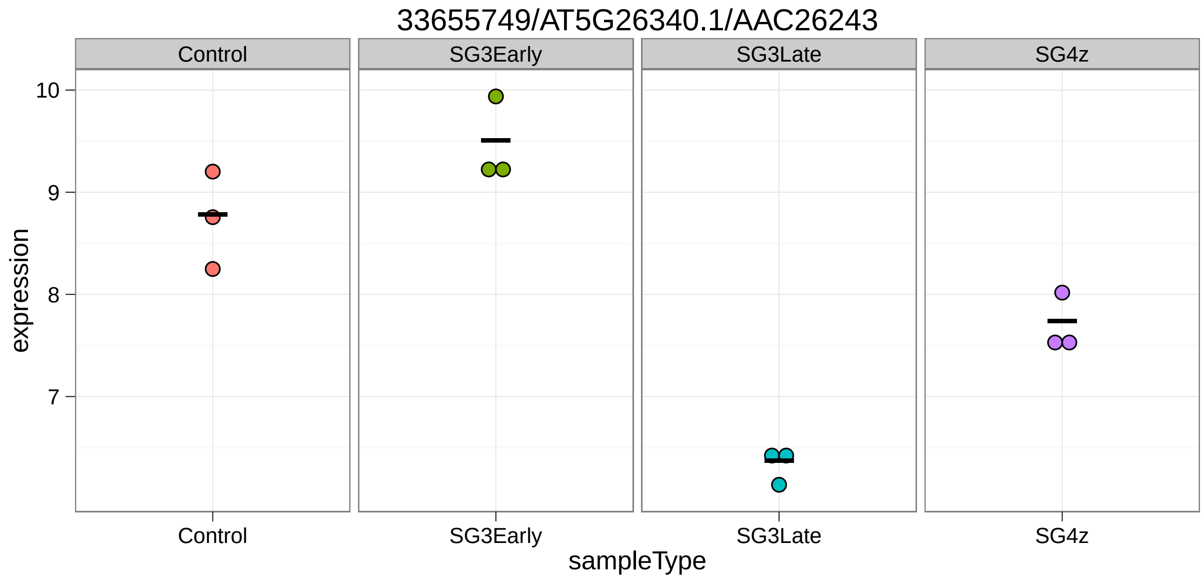

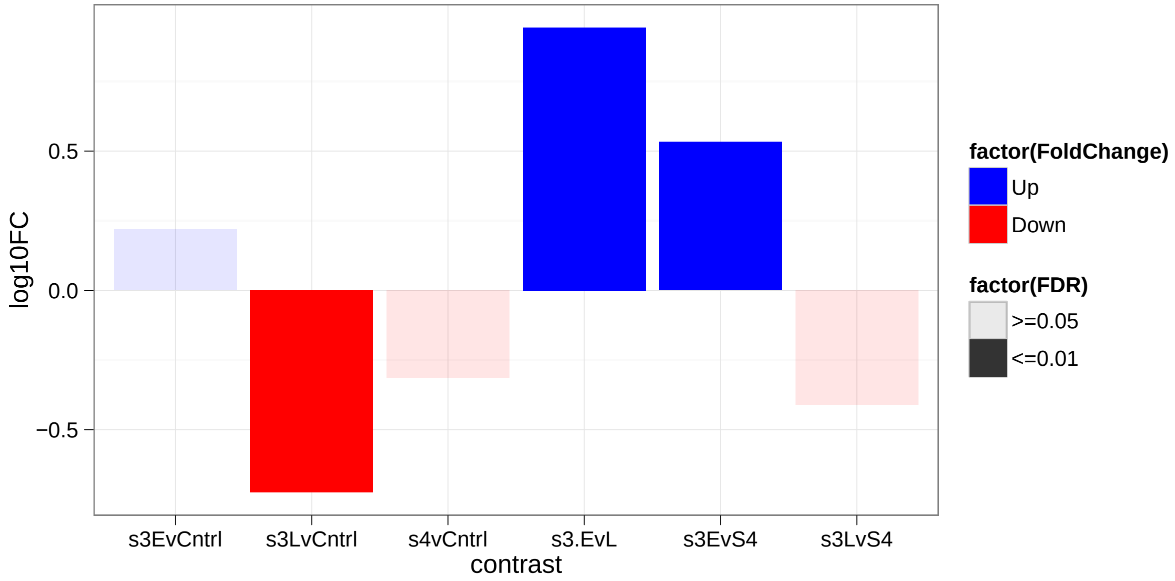

	contrast	logFC	adjPVal
1	s3EvCntrl	0.73	0.34
2	s3LvCntrl	-2.4	0.0011
3	s4vCntrl	-1	0.055
4	s3.EvL	3.1	0.00031
5	s3EvS4	1.8	0.0052
6	s3LvS4	-1.4	0.083

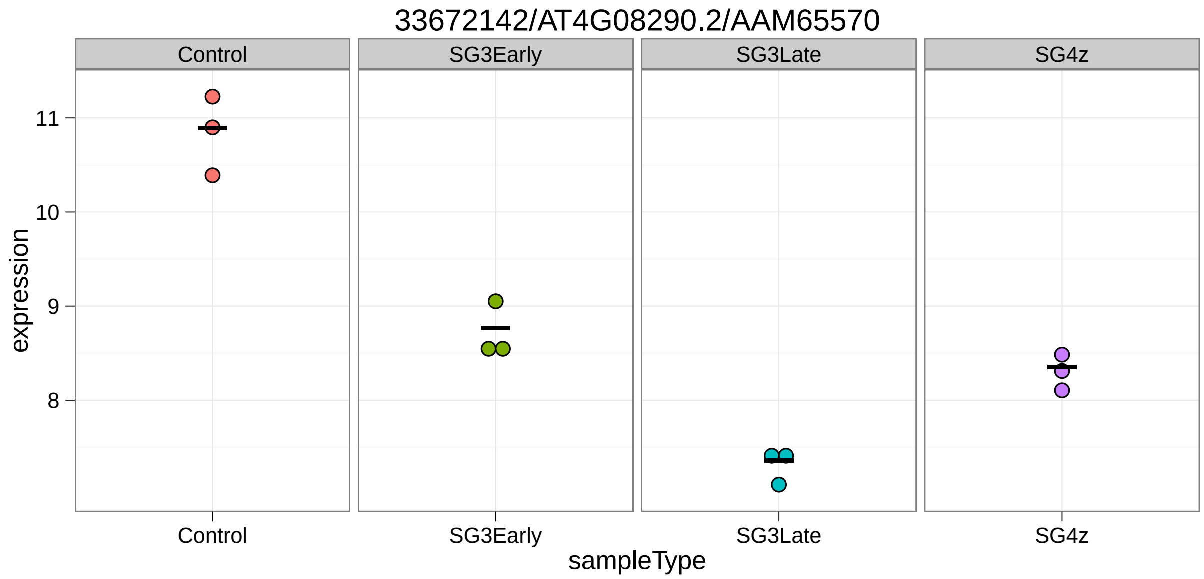

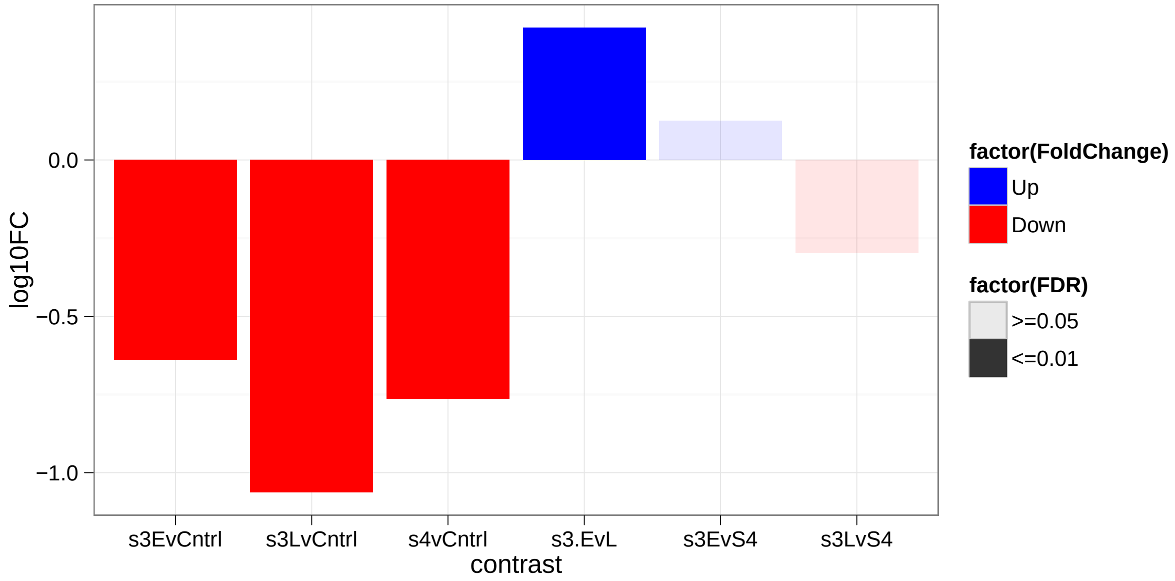

	contrast	logFC	adjPVal
1	s3EvCntrl	-2.1	0.0036
2	s3LvCntrl	-3.5	5.7e-05
3	s4vCntrl	-2.5	0.00024
4	s3.EvL	1.4	0.0065
5	s3EvS4	0.42	0.43
6	s3LvS4	-0.99	0.12

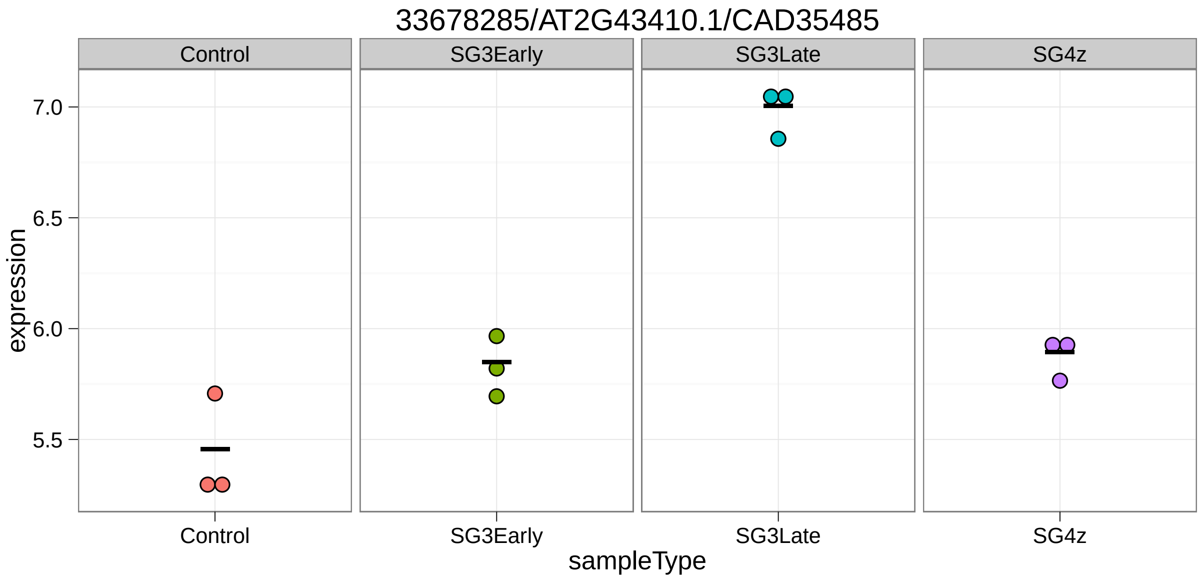

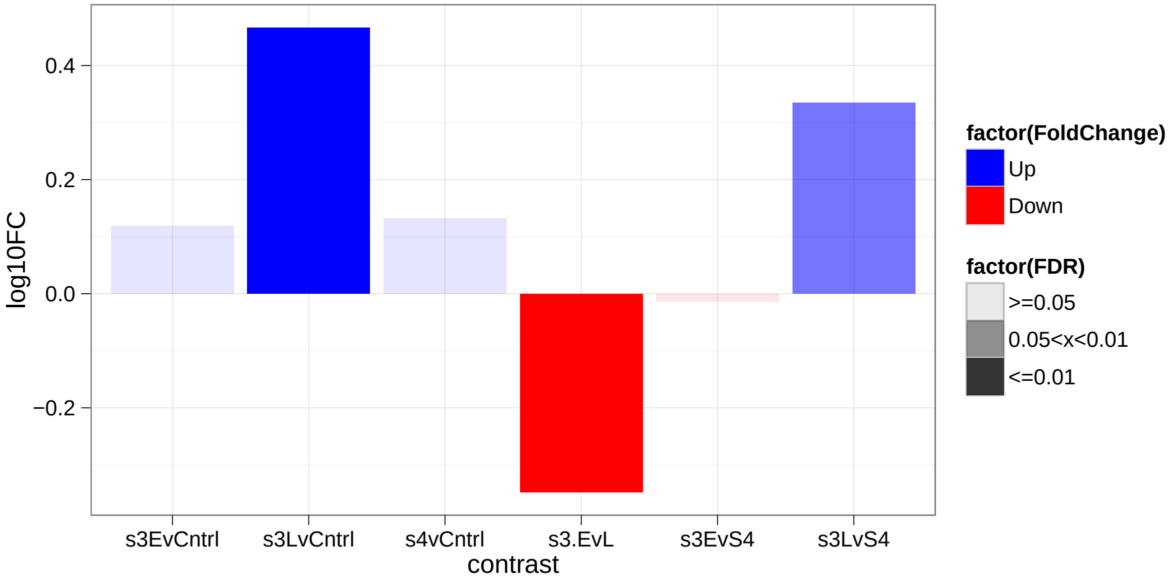

	contrast	logFC	adjPVal
1	s3EvCntrl	0.39	0.32
2	s3LvCntrl	1.5	0.00046
3	s4vCntrl	0.44	0.12
4	s3.EvL	-1.2	0.0019
5	s3EvS4	-0.046	0.93
6	s3LvS4	1.1	0.014

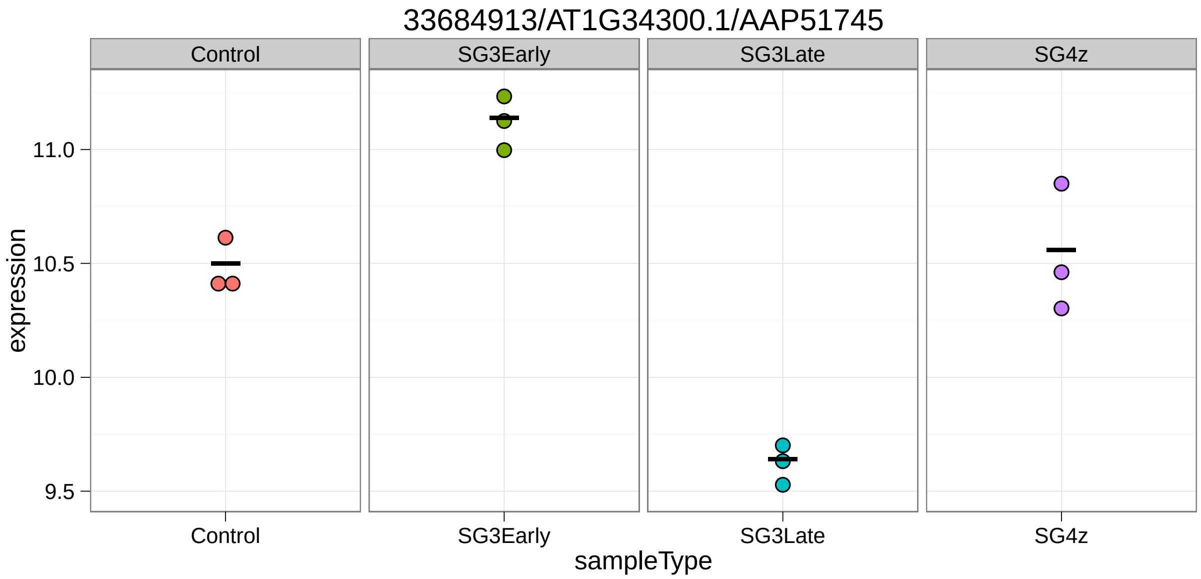

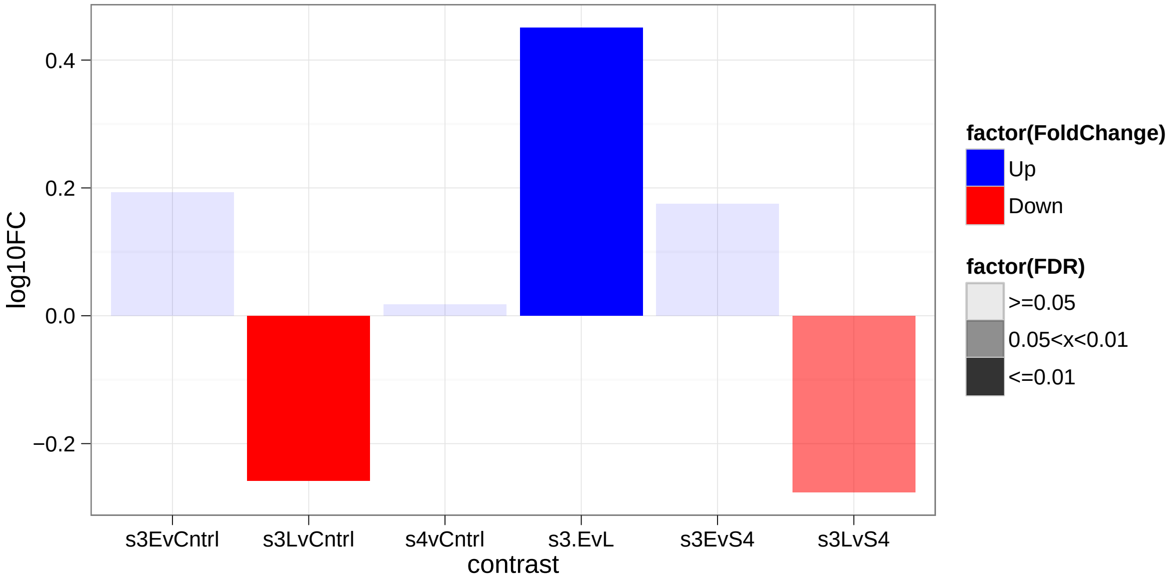

	contrast	logFC	adjPVal
1	s3EvCntrl	0.64	0.13
2	s3LvCntrl	-0.86	0.0089
3	s4vCntrl	0.059	0.9
4	s3.EvL	1.5	0.00071
5	s3EvS4	0.58	0.056
6	s3LvS4	-0.92	0.043
