# Supplementary material for: Global changes in gene expression during compatible and incompatible interactions of cowpea (Vigna unguiculata L.) with the root parasitic angiosperm Striga gesnerioides
Source: BMC Genomics. 2012 Aug 17;13:402. doi: 10.1186/1471-2164-13-402 (PMC3505475; doi:10.1186/1471-2164-13-402)
Supplement: Additional file 7 — GO enrichment SG3 6 dpi. [file 1471-2164-13-402-S7.docx]

| **Categories**  **Additional file 7. Candidate genes from GOterm gene enrichment using Gorilla with p values less than 10-^3^ using differentially expressed GSRs at 5% FDR threshold in cowpea infected with *S.gesnerioides* race 3 at early stage of infection (6 dpi).** | **Sequence ID** | **Annotation** | **Fold change** |
| --- | --- | --- | --- |
|  |  |  |  |
| BIOLOGICAL PROCESS |  |  |  |
| *Programmed cell death* | 33680262 | AT5G46270 - protein binding / transmembrane receptor | 1.91 |
|  | 33663536 | AT5G65380 - ripening-responsive protein, putative | 1.53 |
|  |  |  |  |
| *Cellular processes* | 33694140 | AT4G05230 - ubiquitin family protein | 3.57 |
|  | 33688614 | AT3G21340 - leucine-rich repeat protein kinase, putative | 2.85 |
|  | 33654455 | AT1G56140 - leucine-rich repeat family protein | 2.08 |
|  | 33666862 | AT1G72540 - protein kinase, putative | 1.82 |
|  | 33653892 | AT5G51560 - leucine-rich repeat transmembrane protein kinase, putative | 1.79 |
|  | 33682666 | AT1G07560 - leucine-rich repeat protein kinase, putative | 1.57 |
|  | 33677519 | AT3G62220 - serine/threonine protein kinase, putative | 1.52 |
|  | 33678992 | AT3G63170 - chalcone isomerase | 1.43 |
|  |  |  |  |
| *Osmotic stress* | 33666488 | AT4G37530 - peroxidase, putative | 1.39 |
|  |  |  |  |
| *Apoptosis* | 33680262 | AT5G46270 - protein binding / transmembrane receptor | 1.91 |
|  |  |  |  |
| *Defence response* | 33658423 | AT1G77920 - bzip family transcription factor | 1.63 |
|  |  |  |  |
|  |  |  |  |
| FUNCTION |  |  |  |
| *Hydrolase activity* | 33670460 | AT4G18550 - lipase class 3 family protein | 6.27 |
|  | 33693554 | AT5G44020 - acid phosphatase class b family protein | 1.76 |
|  | 33654737 | AT1G06550 - enoyl-coa hydratase/isomerase family protein | 1.71 |
|  |  |  |  |
| *Catalytic activity* | 33692312 | AT5G01050 - laccase family protein / diphenol oxidase family protein | 4.41 |
|  | 33663803 | AT5G05340 - peroxidase, putative | 3.71 |
|  | 33646588 | AT3G44260 - ccr4-not transcription complex protein, putative | 1.92 |
|  | 33648411 | AT3G15290 - 3-hydroxybutyryl-coa dehydrogenase, putative | 1.78 |
|  | 33654737 | AT1G06550 - enoyl-coa hydratase/isomerase family protein | 1.71 |
|  |  |  |  |
| *Binding* | 33680085 | AT5G23710 - dna binding / dna-directed rna polymerase | 2.48 |
|  | 33689446 | AT2G02160 - zinc finger (ccch-type) family protein | 1.88 |
|  | 33662272 | AT1G35460 - basic helix-loop-helix (bhlh) family protein | 1.81 |
|  | 33653892 | AT5G51560 - leucine-rich repeat transmembrane protein kinase, putative | 1.79 |
|  | 33654666 | AT5G14040 - mitochondrial phosphate transporter | 1.42 |
