# Supplementary material for: Global changes in gene expression during compatible and incompatible interactions of cowpea (Vigna unguiculata L.) with the root parasitic angiosperm Striga gesnerioides
Source: BMC Genomics. 2012 Aug 17;13:402. doi: 10.1186/1471-2164-13-402 (PMC3505475; doi:10.1186/1471-2164-13-402)
Supplement: Additional file 8 — GO enrichment SG3 13 dpi. [file 1471-2164-13-402-S8.docx]

| **Categories**  **Additional file 8. Candidate genes from GOterm gene enrichment using Gorilla with p values less than 10^-3^ using differentially expressed GSRs at 5% FDR threshold in cowpea infected with *S.gesnerioides* race 3 at late stage of infection (13 dpi).** | **Sequence ID** | **Annotation** | **Fold change** |
| --- | --- | --- | --- |
|  |  |  |  |
| BIOLOGICAL PROCESS |  |  |  |
| *Response to inorganic substance* | 33655605 | AT2G19310 - hypothetical protein | 2.97 |
|  | 33653093 | AT4G01280 - myb family transcription factor | 1.67 |
|  | 33667653 | AT1G74840 - myb family transcription factor | 1.65 |
|  |  |  |  |
| *Response to hydrogen peroxide* | 33655605 | AT2G19310 - low molecular weight heat-shock protein | 2.97 |
|  |  |  |  |
| *Response to cadmium ion* | 33648639 | AT2G44060 - late embryogenesis abundant family protein | 1.98 |
|  | 33653093 | AT4G01280 - myb family transcription factor | 1.67 |
|  |  |  |  |
| *Response to bacterium* | 33660824 | AT1G53240 - malate dehydrogenase (nad), mitochondrial | 1.35 |
|  |  |  |  |
| FUNCTION |  |  |  |
| 3-deoxy-7-phosphoheptulonate synthase activity | 33649823 | AT1G22410 - dahp synthetase, putative | -1.81 |
