# Supplementary material for: Global changes in gene expression during compatible and incompatible interactions of cowpea (Vigna unguiculata L.) with the root parasitic angiosperm Striga gesnerioides
Source: BMC Genomics. 2012 Aug 17;13:402. doi: 10.1186/1471-2164-13-402 (PMC3505475; doi:10.1186/1471-2164-13-402)
Supplement: Additional file 9 — GO enrichment SG4z 13 dpi. [file 1471-2164-13-402-S9.docx]

| **Categories**  **Additional file 9. Candidate genes from GOterm gene enrichment using Gorilla with p values less than 10^-3^ using differentially expressed GSRs at 5% FDR threshold in cowpea infected with *S.gesnerioides* race 4z at late stage of infection (13 dpi)** | **Sequence ID** | **Annotation** | **Fold change** |
| --- | --- | --- | --- |
|  |  |  |  |
| BIOLOGICAL PROCESS |  |  |  |
| *Response to inorganic substance* | 33660824 | AT1G53240 - malate dehydrogenase (nad), mitochondrial | -2.48 |
|  | 33674937 | AT4G34050 - caffeoyl-coa 3-o-methyltransferase, putative | -2.48 |
|  | 33653093 | AT4G01280 - myb family transcription factor | 2.13 |
|  |  |  |  |
| *Response to hydrogen peroxide* | 33655605 | AT2G19310 - low molecular weight heat-shock protein | 2.64 |
|  |  |  |  |
| *Response to metal ion* | 33688216 | AT1G70730 - phosphoglucomutase, cytoplasmic, putative | -2.72 |
|  | 33675396 | AT1G23190 - phosphoglucomutase, cytoplasmic, putative | -2.50 |
|  | 33674937 | AT4G34050 - caffeoyl-coa 3-o-methyltransferase, putative | -2.48 |
|  | 33649527 | AT2G01140 - fructose-bisphosphate aldolase, putative | -2.10 |
|  |  |  |  |
| *Response to cadmium ion* | 33688216 | AT1G70730 - phosphoglucomutase, cytoplasmic, putative | -2.72 |
|  | 33649527 | AT2G01140 - fructose-bisphosphate aldolase, putative | -2.10 |
|  |  |  |  |
| *Defense response to bacterium* | 33660824 | AT1G53240 - malate dehydrogenase (nad), mitochondrial | -2.48 |
|  |  |  |  |
|  |  |  |  |
| COMPONENT |  |  |  |
| *Cell wall* | 33654553 | AT2G45220 - pectinesterase family protein | -5.62 |
|  | 33676062 | AT3G47400 - pectinesterase family protein | -3.90 |
|  | 33647292 | AT5G10840 - endomembrane protein 70, putative | -1.91 |
|  |  |  |  |
| *Plasma membrane* | 33672271 | AT3G27960 - kinesin light chain-related | -3.82 |
|  | 33665952 | AT4G36750 - quinone reductase family protein | -3.53 |
|  | 33691443 | AT4G26540 - kinase | -3.15 |
|  | 33678202 | AT5G01020 - protein kinase family protein | -2.02 |
|  | 33649242 | AT1G75500 - nodulin mtn21 family protein | -1.77 |
|  | 33680127 | AT1G64760 - glycosyl hydrolase family 17 protein | -1.54 |
|  | 33652975 | AT4G21450 - vesicle-associated membrane family protein | -1.63 |
|  |  |  |  |
| *Plant-type cell wall* | 33668001 | AT1G02810 - pectinesterase family protein | -13.62 |
|  | 33654553 | AT2G45220 - pectinesterase family protein | -5.62 |
|  | 33693005 | AT1G78060 - glycosyl hydrolase family 3 protein | -4.25 |
|  | 33647292 | AT5G10840 - endomembrane protein 70, putative | -1.91 |
