# Supplementary material for: Global changes in gene expression during compatible and incompatible interactions of cowpea (Vigna unguiculata L.) with the root parasitic angiosperm Striga gesnerioides
Source: BMC Genomics. 2012 Aug 17;13:402. doi: 10.1186/1471-2164-13-402 (PMC3505475; doi:10.1186/1471-2164-13-402)
Supplement: Additional file 10 — Primer sequences for qRT-PCR. [file 1471-2164-13-402-S10.docx]

**Additional file 10. Primer sequences used for quantitative qRT-PCR**

| **Sequence ID** | **Forward primer** | **Reverse primer** | **Amplicon length [bp]** |
| --- | --- | --- | --- |
| 33646940 | TGATGATGCAGCTTCTCC | GACTCGTGCTTGAGCTTC | 155 |
| 33656017 | TGGAGAAGGGTAGCAGTGTG | GCCGTCCAAGTAGCATCAAT | 160 |
| 33693491 | TGCATTCCATTCCTCAATCA | TGGAGGAAATTGAAGCAAAGA | 149 |
| 33662461 | ATGGTGGTTCCACTGACTC | GTTCACCCTTCTGCAGTTC | 152 |
| 33675942 | TGGCAGTGGCAATAGATTCA | TCACAAGAGAAACCGATGGA | 144 |
| 33677660 | CATGCTTTGGACGCTCTC | ACCAGCCTTCGCCATTTC | 148 |
| 33677650 | ATGATGGCGGTGTTCTGAAG | TGAACCTCCGGTTGAGTTTG | 150 |
| 33681428 | AGGTCTTCGACAATATGGG | GGAAACTTTCACAGATGGAG | 118 |
| 33659812 | TCAAGTGCTTGTGAATGTGT | CTCCTTACCCAGAAACCTCT | 94 |
|  |  |  |  |
